# Use of historical isoscapes to develop an estuarine nutrient baseline

**DOI:** 10.3389/fmars.2023.1257015

**Published:** 2023-09-06

**Authors:** Lena K. Champlin, Andrea Woolfolk, Autumn J. Oczkowski, Audrey Rittenhouse, Andrew B. Gray, Kerstin Wasson, Farzana I. Rahman, Paula Zelanko, Nadine B. Quintana Krupinski, Rikke Jeppesen, John Haskins, Elizabeth B. Watson

**Affiliations:** 1 Department of Biodiversity, Earth & Environmental Sciences and the Academy of Natural Sciences of Drexel University, Philadelphia, PA, United States; 2 Elkhorn Slough National Estuarine Research Reserve, Royal Oaks, CA, United States; 3 U.S. Environmental Protection Agency (EPA), Atlantic Ecology Division, Narragansett, RI, United States; 4 Department of Environmental Sciences, University of California Riverside, Riverside, CA, United States; 5 Ecology and Evolutionary Biology, University of California Santa Cruz, Santa Cruz, CA, United States; 6 Department of Earth and Planetary Sciences, University of California Santa Cruz, Santa Cruz, CA, United States; 7 Department of Ecology and Evolution, Stony Brook University, Stony Brook, NY, United States

**Keywords:** nitrogen, eutrophication, stable isotopes, isoscapes, sediment cores, baseline

## Abstract

Coastal eutrophication is a prevalent threat to the healthy functioning of ecosystems globally. While degraded water quality can be detected by monitoring oxygen, nutrient concentrations, and algal abundance, establishing regulatory guidelines is complicated by a lack of baseline data (e.g., pre-Anthropocene). We use historical carbon and nitrogen isoscapes over ~300 years from sediment cores to reconstruct spatial and temporal changes in nutrient dynamics for a central California estuary, Elkhorn Slough, where development and agriculture dramatically enhanced nutrient inputs over the past century. We found strong contrasts between current sediment stable isotopes and those from the recent past, demonstrating shifts exceeding those in previously studied eutrophic estuaries and substantial increases in nutrient inputs. Comparisons of contemporary with historical isoscapes also revealed that nitrogen sources shifted from a historical marine-terrestrial gradient with higher δ^15^N near the inlet to amplified denitrification at the head and mouth of the modern estuary driven by increased N inputs. Geospatial analysis of historical data suggests that an increase in fertilizer application – rather than population growth or increases in the extent of cultivated land – is chiefly responsible for increasing nutrient loads during the 20^th^ century. This study demonstrates the ability of isotopic and stoichiometric maps to provide important perspectives on long-term shifts and spatial patterns of nutrients that can be used to improve management of nutrient pollution.

## 1 Introduction

Coastal eutrophication, resulting from anthropogenic nutrient inputs, is an increasing threat to the healthy functioning of ecosystems (Nixon, 1995; Duarte et al., 2009). Coastal watersheds support greater than half of the world’s population (Cloern et al., 2016) leading to rapid development in urban, agricultural, and industrial coastal areas (Rabalais et al., 2009; Paerl et al., 2014). Fossil fuel combustion, land clearing, inadequate wastewater treatment, and inputs of fertilizer or agricultural waste increase macronutrients (carbon, nitrogen, phosphorus) which fuel enhanced productivity and respiration (Nixon, 1995; Cloern, 2001). Nitrogen pollution is problematic as it alters the equilibrium between production and metabolism in the coastal zone and is associated with negative impacts on water quality (Conley et al., 2009; Camacho-Cruz et al., 2022) including development of hypoxia (Howarth et al., 2011), blooms of opportunistic algae (Teichberg et al., 2010; Cabanillas-Terán et al., 2019), loss of valuable seagrass (Duarte, 1995) and coastal wetlands (Deegan et al., 2012).

Anthropogenic eutrophication is recognized as an issue of societal concern (Nixon, 1995), however successful management of nutrient pollution is still in its infancy because of the complexity of local drivers of eutrophication, identification of nutrient sources, and challenges of regulating non-point sources (Porter et al., 2015). For example, nutrient cycling in estuaries is regulated by site-specific conditions including estuarine circulation patterns riverine inputs, climate seasonality, and suspended sediment concentrations, in addition to anthropogenic nutrient inputs. Among estuaries throughout the U.S., differences are observed in the degree of physical vs. biological processes controlling ecosystem metabolism (Caffrey, 2003). Furthermore, in coastal upwelling zones, ocean water advected with tides can be the dominant nutrient source to estuaries (White et al., 2013). Due to these complexities, nutrient abatement often does not lead to linear and predictable improvements in water quality (Duarte et al., 2009). In addition, non-point source nutrient pollution, including agricultural runoff, has proved difficult to regulate (Porter et al., 2015). These management issues point to the importance of considering nutrient reduction goals that are informed by scientific understanding of historical changes in nutrients.

To guide nutrient reduction goals, scientific studies have used sediment cores to reconstruct nutrient baselines and historical trajectories. In lakes, organism assemblages (primarily insects and diatoms) in dated sediment cores have been interpreted as indicators of eutrophication (Battarbee, 1999). These studies uncovered the magnitude and timing of human alterations, and permitted quantitative reconstruction of specific nutrients concentrations to identify baseline values for regulatory efforts (Bennion et al., 2005). In coastal and estuarine environments, paleoenvironmental reconstructions from sediment cores have played important roles in shaping our understanding of nutrient pollution, especially in Chesapeake Bay and south Florida where large-scale efforts have focused on reversing the negative impacts of nutrients (Willard and Cronin, 2007). Sedimentary analyses of geochemical and biological proxies include redox sensitive metals as anoxia indicators (Cooper and Brush, 1991; Boothman and Coiro, 2009), microfossil assemblages as indicators of inputs (Cooper, 1995; Jensen et al., 1999), and carbon, nitrogen, and phosphorus accumulation records as indicators of benthic organic matter (Zimmerman and Canuel, 2002; Engstrom et al., 2006).

Both nitrogen (N) and carbon (C) stable isotopes of sedimentary matter can provide a useful perspective on estuarine nutrient dynamics (Oczkowski et al., 2011). Carbon and nitrogen have two common stable isotopes (${}_{6}^{12}\text{C}$, 98.93%; ${}_{6}^{13}\text{C}$, 1.07%; ${}_{7}^{14}\text{N}$, 99.63%; ${}_{7}^{15}\text{N}$, 0.37%). Chemical, physical, and biological processes often discriminate between the two stable isotopes, leading to measurable differences in ^15^N/^14^N and ^13^C/^12^C ratios among different chemical pools or biota (Post, 2002; Sigman et al., 2009). Sedimentary stable N isotopes, in concert with stable C isotopes and stoichiometric ratios, can distinguish between marine and terrestrial sources of nutrients Peters et al., 1978; Schubert and Calvert, 2001; Eerkens et al., 2013). For example, previous studies traced sea to land gradients of nitrate source using δ^15^N values in a Californian estuary and coastal lagoon, which showed higher δ^15^N values in macrophytes near the marine endmember (δ^15^N ~ 10 to 12‰) and lower near terrestrial inputs (δ^15^N ~ 6 to 8‰) (Huntington and Boyer, 2008; Carriquiry et al., 2016). Furthermore, δ^13^C and C/N ratios also display a marine-terrestrial gradient because phytoplankton productivity, among other factors, produces higher δ^13^C (~ −18 to −25 ‰) and lighter C/N (~14 to 24) signatures compared to terrestrial plants (Cloern et al., 2002; Carriquiry et al., 2016). In addition to nutrient source, stable isotopes may reflect relative loads. For example, denitrification imparts a large isotope effect (^15^N discrimination), consequently, aquatic ecosystems with large nitrate inputs and significant rates of denitrification have high δ^15^N values (Anderson and Cabana, 2005; Oczkowski et al., 2008).

In this study, we analyzed historical baseline shifts in sedimentary N isotopes, C isotopes, and C/N stoichiometric ratios, but added an explicitly spatial dimension to our investigation through the construction of contemporary and historical whole-estuary isoscapes. The term isoscape refers to any spatial representation of isotope data (West et al., 2008). Isoscapes are established in earth system research (Bowen, 2010) and emerging in paleoenvironmental studies (Reade et al., 2023). Their use has been applied to a variety of spatiotemporal questions to identify spatial patterns in N fixation (Hellmann et al., 2016), fossil fuel combustion (Bowen 2010), variability in river water sources (Brooks et al., 2012), as well as for tracking migratory animals (Hobson et al., 2009). In the context of coastal and estuarine science, isoscapes are used by monitoring programs to identify water pollution hotspots and transitions between eutrophic and oligotrophic waters (Kendall et al., 2010; Radabaugh et al., 2013; Watson et al., 2018; Sánchez et al., 2023), in mussels to identify circulation patterns (Vokhshoori and McCarthy, 2014; Vokhshoori et al., 2014), and in archeological research to identify diets (Eerkens et al., 2013).

We assembled current and historical whole-estuary N and C isoscapes, as well as C/N stoichioscapes across the spatial extent of an estuary in central California. Samples from 85 sediment cores collected across the estuary reconstructed isoscapes and stoichioscapes from six time periods (ca. 1726-2010), and higher resolution analysis was performed on six focal cores to create timeseries. Chronological control was provided via ^14^C, ^137^Cs, and ^210^Pb radiometric dating. Increased nutrient inputs over time were attributed to watershed sources by comparing agricultural fertilization, wastewater, livestock, and nitrogen deposition using a geospatial model of nitrogen inputs. Our findings reveal unprecedented insights into the timing and sources of nutrients and provide a valuable long-term perspective on nutrient pollution where water quality improvements are a priority for coastal managers.

## 2 Materials and methods

### 2.1 Study site

**FIGURE 1:**
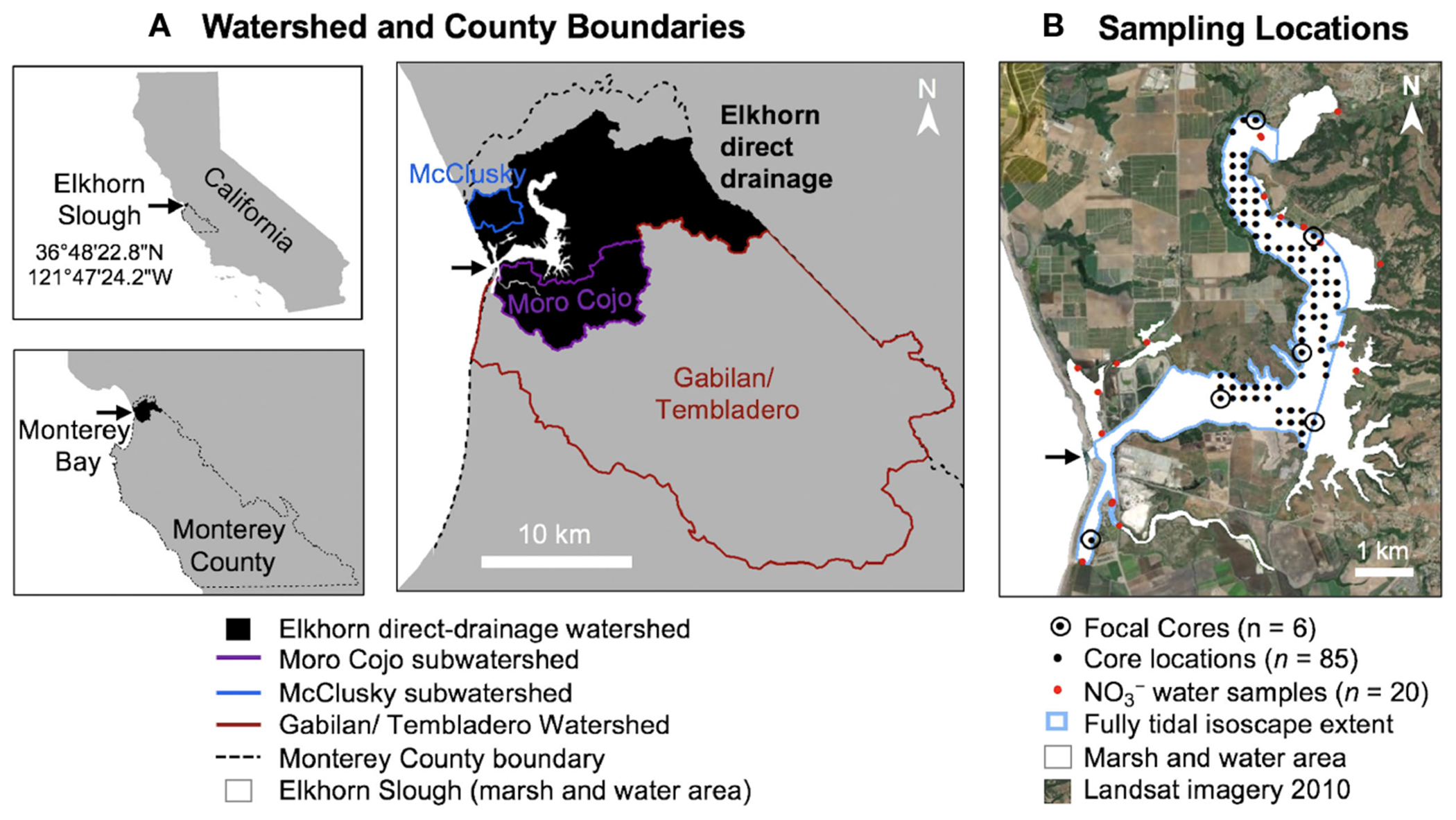
**(A)** Map showing watersheds within Monterey County contributing to Elkhorn Slough. The Elkhorn Watershed (black shaded), including Moro Cojo and McClusky subwatersheds, was used for our NLM analysis because it was defined as the direct watershed in local reports and resources (Dickert and Tuttle, 1985; ESNERR and ESF, 2021). **(B)** Core sampling locations within Elkhorn Slough and water quality monitoring stations (Esri World Imagery acquired 2010).

Elkhorn Slough is a shallow (3.5 m mean depth below mean lower low water or MLLW), marine (salinity ~ 30ppt), tidal (1.7m mean diurnal tidal range) estuary located in along Monterey Bay in Central California (USA) (Figure 1). The estuary is part of several protected areas. All tidal lands below the mean high water (MHW) mark are part of the Monterey Bay National Marine Sanctuary (Gee et al., 2010) and adjacent lands are mostly protected open space including the 704 ha Elkhorn Slough National Estuarine Research Reserve. The 10-km main channel of the Slough is bordered by extensive intertidal salt marshes and mudflats, and several shallow water impoundments are associated with water control structures that restrict tidal exchange beneath railroad tracks along the eastern border of the estuary. Maximum daily tidal excursion distances are a considerable fraction of the length of the slough (5-7 km) and the tidal prism is comparable to the volume of the estuary below MLLW (Monismith et al., 2005; Nidzieko and Monismith, 2013). Physical forcing, including tidal exchange and runoff volume, impart a strong influence on nutrient patterns in the modern estuary (Caffrey et al., 2007). Historical sources from the 19^th^ century suggest that the tidal exchange was extensive, including the adjacent estuaries of the Old Salinas River channel, Moro Cojo and Tembladero Sloughs, but a partial sandbar at the shared marine inlet presumably damped tidal exchange, with seasonal variation. During the early 20^th^ century, sedimentation near the inlet was caused by erosion of cleared land in the watershed and diking of large parts of the adjacent, interconnected estuary, which decreased the tidal prism. However, construction of a marine inlet for a deepwater harbor in 1946 then dramatically increased tidal exchange to the Slough (Watson et al., 2019; Woolfolk, unpublished data).

The Elkhorn direct-drainage watershed, defined here including the Moro Cojo and McClusky subwatersheds (Figure 1A), has an estimated surface area of 182 km^2^ (Silberstein et al., 2002). Nitrate from the Carneros Creek at the head of the estuary enters as intermittent pulses of runoff from high rainfall events (Caffrey et al., 2007). Additionally, the estuary receives inputs from the larger Gabilan/Tembladero watershed to the south and occasionally from the Salinas watershed through a lift gate, which both flow into the Old Salinas River channel and enter near the marine mouth of Elkhorn Slough (Johnson et al., 2007). A total maximum daily load (TMDL) addressing nitrogen for the Salinas River was published (Osmolovsky et al., 2013) and accepted by the EPA in 2015. However, a TMDL for the direct-drainage watershed is currently in progress (Sutula et al., 2022). Therefore, this study contributes detailed analysis of the terrestrial sources in the direct Elkhorn watershed that extends prior to other watershed models. Approximately 26% of the combined watersheds receive extensive fertilizer applications associated with row crop agriculture (Chapin et al., 2004). The mild climate allows two to three harvests per year, which leads to especially high fertilizer application (Johnson et al., 2007). While cattle operations have historically been prevalent in the watershed (>3300 individuals in 1970), the number of cows has declined to close to 1000 individuals in 2010, with most of the current population restricted to Moon Glow Dairy which uses a nutrient retention pond (United States Department of Agriculture, 1850-2012; Silberstein et al., 2002).

### 2.2 Sediment core collection

Eighty-five ~ three-meter-deep sediment cores were collected during 2010 from the vertices of a 200 m x 200 m grid superimposed over the tidal and never-diked portions of the estuary (Figure 1B). Most of the sediment cores were collected using a Russian peat borer to minimize compaction; in a few locations, a vibracorer was necessary to penetrate sands. Six focal cores (including the Yampah core collected in 2004; Watson et al., 2011) were selected for high-resolution analyses and were collected using a piston corer with polycarbonate liners to obtain intact core sections for scanning and archiving. Focal cores were split into 1-cm sections; the remaining cores were sectioned into 10 cm intervals for 0-50 cm depths, and into 25-cm intervals for 50-100 cm depths. Core splits were archived at the LacCore repository at the University of Minnesota.

### 2.3 Age-depth chronology

Chronologies were created using downcore profiles of ^210^Pb, ^137^Cs, and ^226^Ra measured with a low energy germanium multichannel gamma spectrometer. Historical geochemical markers included Pb concentrations measured using ICP-AES following four-acid extractions (Kemp et al., 2012), AMS radiocarbon dating of fossil peat (García-García et al., 2013), and magnetic susceptibility and imaging using a Geotek Multi-Sensor core logger. The certified reference standard for analysis of Pb concentration was NIST San Joaquin Soil (Percentage recovery = 85%). The maximum depth of radiocesium was assigned an age of 1953, radiocesium peaks were assigned an age of 1963, and total lead concentration peaks were assigned an age of 1974.

**FIGURE 2:**
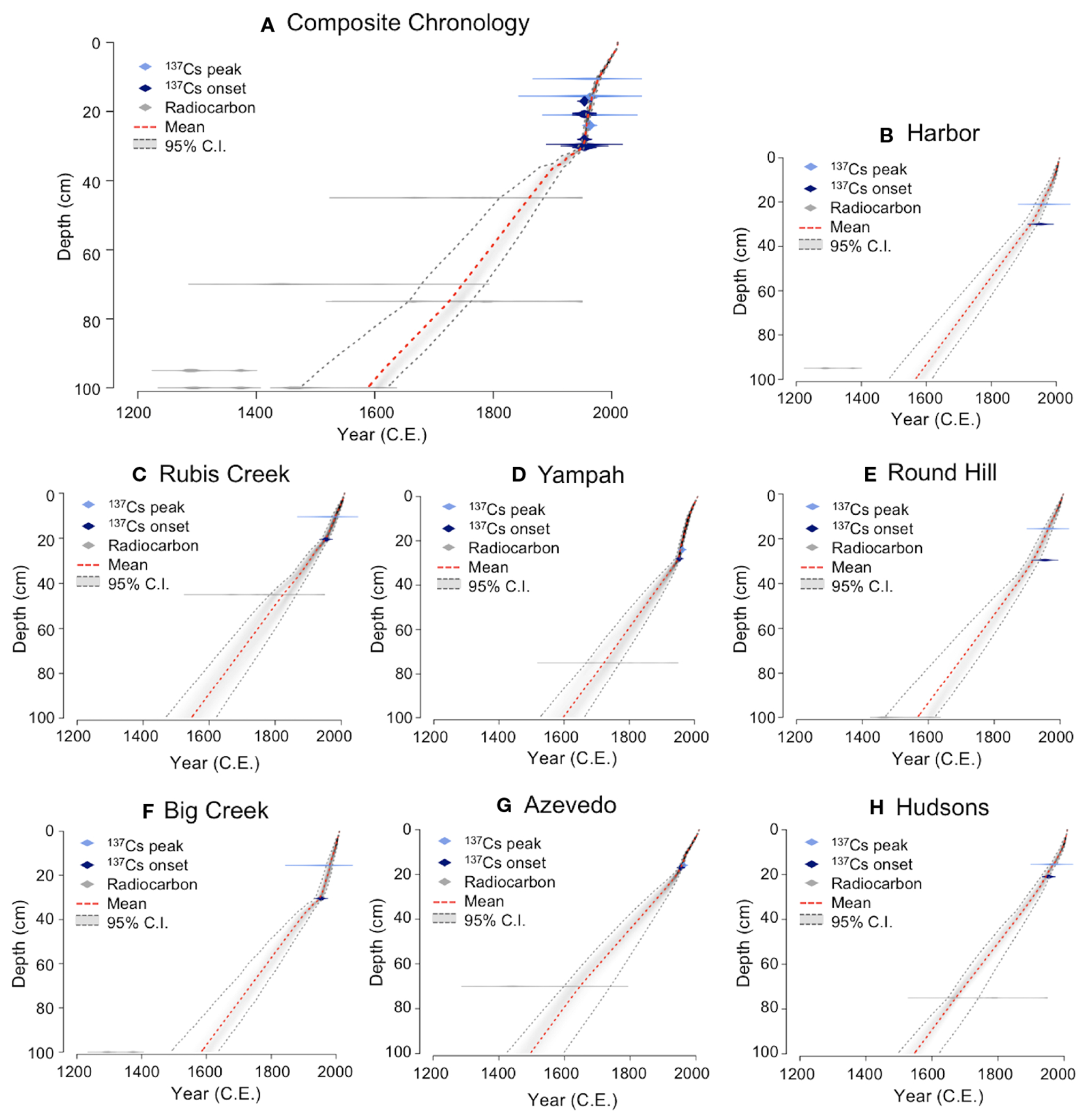
Age-depth relationships from seven coring sites along the spatial gradient of Elkhorn Slough arranged from mouth to head **(A)** A composite chronology for all seven coring sites; **(B)** Harbor; **(C)** Rubis Creek; **(D)** Yampah; **(E)** Round Hill; **(F)** Big Creek; **(G)** Azevedo; and **(H)** Hudsons. The chronologies were created using ^210^Pb *Plum* models (R Package “Plum” version 0.2.2; Blaauw et al., 2021), which incorporate lead, radiocesium, and radiocarbon dating.

Lead-210, radiocesium, and radiocarbon dating were combined in an age-depth model using a Bayesian approach to construct chronologies for seven cores (including the six focal cores and an additional core Azevedo collected in 2004; Watson et al., 2011). The age model ^210^Pb *Plum* (R Package “rplum” version 0.2.2; Blaauw et al., 2021) in R version 4.0.5 uses the same statistical approach as the previous model Bacon (Blaauw and Christen, 2011), but incorporates radionuclide dating including parameters of deposition of ^210^Pb, supported ^210^Pb, and accretion rates. The *Plum* model was selected because it can account for incremental ^210^Pb data over depth in the cores, as opposed to using the analytical approach of the continuous rate of supply model. Additionally, this model has been used previously for chronologies of estuarine sediments (Wigand et al., 2021). Within Elkhorn Slough, sediment accumulation rates varied little from site to site over the past century and were similar to values reported previously (Schwartz et al., 1986; Hornberger, 1991; Watson et al., 2011); thus, to estimate ages for the 85 undated cores, we compiled a composite core chronology using the seven cores to represent mean age-date model for the entire estuary (Figure 2). This composite core chronology was then applied to the 85 undated cores, using the composite age-depth relationship to estimate dates for the depth segments utilized for isotopic and stoichiometric measurements. We report the mean year output of the model and 95% confidence intervals around the mean (Table S4) (Stuiver and Polach, 1977; Reimer et al., 2020).

### 2.4 Isotopic and stoichiometric analysis

For the six high-resolution focal sites, cores were analyzed at 1-cm increments (for 0 to 50 cm depths) for stable carbon and nitrogen isotopic composition using a Finnegan Delta Plus continuous flow isotope ratio mass spectrometer (CF-IRMS) using standard methods (McClelland et al., 1997; McKinney et al., 2001), and for carbon and nitrogen concentration using a Flash 1112 EA. For the 85 coarser resolution cores, sediments were analyzed for carbon and nitrogen abundance and stable isotope ratios using a Vario Cube elemental analyzer interfaced to an Isoprime 100 IRMS. Isotope ratios for carbon and nitrogen are reported in permille notation as: $\delta^{a}X=(\frac{R_{\mathrm{sample}}}{R_{\mathrm{standard}}}-1)\times 1000‰$ where *R* is the abundance ratio of the less common (*a*) to more common isotope (Kendall et al., 2010). The reference standard for nitrogen is atmospheric nitrogen gas; the standard for carbon is PeeDee Belemnite; by definition standards have δ=0. The internal working standard for isotopes was blue mussel (δ^15^N = 11.22; δ ^13^C = −18.33). The standards for C/N analysis were cysteine and acetanilide. Sediments were not pretreated to remove inorganic carbon, as acidification did not quantitatively shift ratios. Carbon percent relative to nitrogen percent for the focal cores were compared (Figure S2).

### 2.5 Isoscape and stoichioscape mapping

Whole estuary isoscape and stoichioscape maps were produced using sedimentary stable isotope (δ^13^C and δ^15^N) and molar nutrient stoichiometric (C/N) ratios interpolated from the 85 core locations using ordinary kriging in ArcGIS version 10.2.2 (ESRI, Redlands, CA, USA) to the spatial extent of cored areas in Elkhorn Slough. Maps were created for six depth intervals dated using the composite chronology (ca. 1726-1839, 1839-1885, 1885-1951, 1951-1963, 1963-1981, and 1981-2010) corresponding to sampling which integrated 10-cm depth intervals for the 85 cores. Different interpolation variogram models including spherical, circular, exponential, Gaussian, linear interpolation with linear drift, and linear with quadratic drift were tested. Leave-one-out cross validation of 15% of the points was used to choose the model which yielded the smallest root mean square error between predicted and actual values (Table S5). To ensure that historical differences in interpolation maps were a function of data differences rather than variogram methodology, the spherical kriging method was used for all timepoints. We also applied data from monthly water quality sampling collected by a volunteer-based monitoring program at a network of (~26) stations across Elkhorn Slough since 1988 (Ritter et al., 2008; Gee et al., 2010). Monthly nitrate data from the sites were averaged during the period 1990 to 2010 and mapped using ordinary kriging for comparison to spatial patterns of the isoscape and stoichioscape maps.

### 2.6 Temporal analysis of isotopes and stoichiometry

Trends in isotopic and stoichiometric signatures since the 1850s were examined for the six high-resolution cores. Timeseries analysis of the high-resolution data investigated the statistical significance of trends during the period of increasing fertilizer application, as well as offsets in the signatures associated with the timing of marine inlet construction for the harbor. Statistically significant change points in the timeseries were determined using the Pettitt Test (R Package “trend” version 1.1.4; Pohlert, 2020), a nonparametric test that identifies the year of a step change and assigns significance to the selection (Pettitt, 1979). Datasets of δ^15^N, δ^13^C, and C/N for each of the six high-resolution coring sites were separately tested for the period 1850-2010 (n = 45 time points each). Next, the timeseries were split at the significant step change points that were statistically identified, forming two datasets “before” and “after” the year of change. Trend analysis was performed using linear regression on the split datasets, to model the slope after the split as well as the difference of y-intercept at the step change year (Figure S1 diagrams the slope and intercept of our statistical models). The difference of y-intercept at the step change year is interpreted as an offset in the timeseries, consistent with construction of the harbor inlet when the step occurred at the same time as the construction (1946 ± 10 years). The slope after this step change year is attributed to increasing fertilizer addition to the watershed from 1940-1980.

To compare sediment isotope results to dissolved nutrient concentrations, we compared water quality data from the volunteer monitoring program to the high-resolution sediment cores during a 20-year period. Monthly water sampling of parameters (including salinity) was measured at the sites, and water samples were also collected into brown Nalgene bottles; stored on ice; filtered; and analyzed for nutrients, including nitrate (NO_3_^−^), within 48 hours, or frozen for later analysis in accordance with standard methods (Gee et al., 2010; NOAA National Estuarine Research Reserve System, 2017). Three of the high-resolution sediment cores were collected at the same locations as water quality monitoring sites. For these three water quality sampling stations (Portero Road North, Kirby Park, and Hudsons Landing West), we compared annual mean water column dissolved NO_3_^−^ (μM) and salinity (ppt) to sedimentary δ^15^N values during the same year from 1990-2010.

### 2.7 Historical nitrogen sources

To examine interrelationships between nitrogen pollution and anthropogenic sources over the past century, we parametrized a model of nitrogen inputs to the watershed. Our model was based on the Nitrogen Loading Model (NLM), a geospatial tool to estimate nitrogen inputs to estuaries based on atmospheric deposition, land cover, and wastewater inputs (Valiela et al., 1997; Bowen et al., 2007). The model has performed well when compared to other commonly used water quality models (e.g., SPARROW; Valiela et al., 2000; Latimer and Charpentier, 2010) and is applicable to bodies of water such as Elkhorn Slough underlain by unconsolidated sedimentary deposits and watersheds containing mixes of residential, agricultural, and forest land covers (Kinney and Valiela, 2011).

We applied the NLM model to calculate watershed sources of nitrogen over time in decadal increments from 1930-2010. Elkhorn watershed delineation was based on local reports (Dickert and Tuttle, 1985), and the Elkhorn Slough Reserve mapping resources (ESNERR and ESF, 2021). We compiled historical data on changes in human population from census data (Census of the United States, 1930; 1960; Manson et al., 2012), atmospheric deposition (Viers et al., 2012), homes with wastewater treatment (Zillow Inc, 2021), the areal extent of cultivated and natural lands and impervious surface cover (Dickert and Tuttle, 1985; U.S. Geological Survey (USGS), 2000-2014), and estimated changes in fertilizer application rates in the Elkhorn watershed (based on annual “Commercial Fertilizers” and “Fertilizing Materials” reports published by the California Department of Agriculture, 1925-2012). A full list of parameters and data sources used in the model can be found in the Supplementary Material (Tables S1–S3).

## 3 Results

### 3.1 Age-depth chronology

**FIGURE 3:**
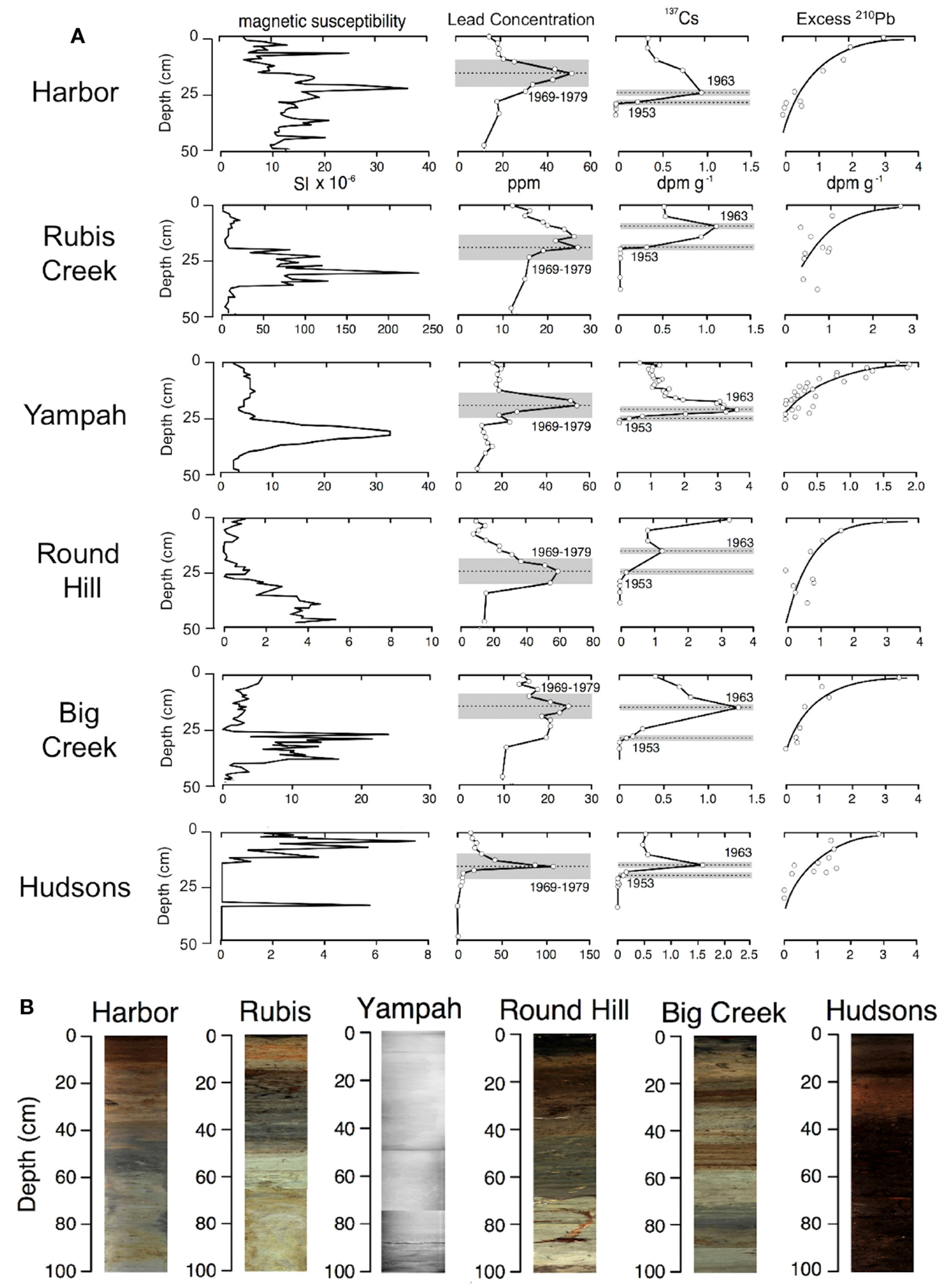
Downcore profiles (showing the top 0-50cm) of **(A)** magnetic susceptibility, total lead concentration (ppm), ^137^Cs activity (dpm g^−1^), and excess ^210^Pb activity (dpm g^−1^). Cores are arranged vertically from the mouth of the estuary (at top), to the head of the estuary (at bottom). **(B)** Images of cores are shown at the bottom; the Yampah core was x-rayed but not visually imaged.

Core chronologies constructed using ^210^Pb, ^137^Cs, and AMS radiocarbon dating showed good agreement for the seven coring sites. Magnetic susceptibility peaks were observed between 30 and 40 cm depths for five of the coring sites (Figure 3). While undated, this peak likely represents watershed erosion associated with early European settlement and is present in stratigraphic records from nearby watersheds (Watson and Byrne, 2012). Elevated concentrations of total lead were recognized in downcore geochemical profiles of six cores collected from Elkhorn Slough (Figure 3). Peaks in lead concentration occurred at depths ranging from 15-26 cm. Especially high concentrations (100+ ppm) were observed for the Hudsons Landing site, which is<300 m from a busy road. Radiocesium peaks were apparent in sediment cores at depths ranging from 10 to 24 cm of depth, whereas basal radiocesium depths ranged from 17 to 30 cm (Figure 3).

### 3.2 Isoscape and stoichioscape mapping

**FIGURE 4:**
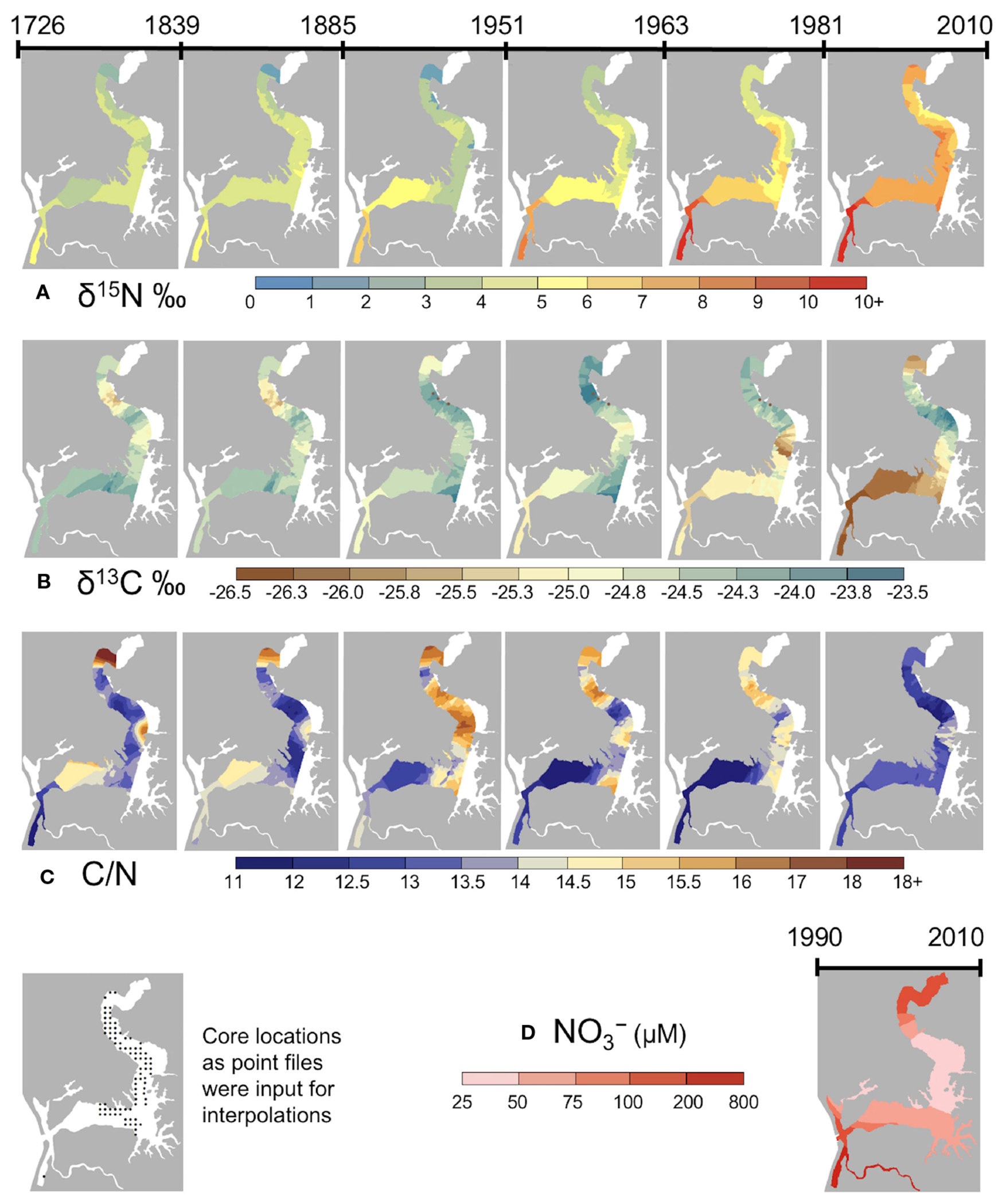
Maps showing historical isoscapes and stoichioscapes across the tidal, never-diked, portion of Elkhorn Slough: **(A)** δ^15^N; **(B)** δ^13^C; **(C)** C/N ratio from ca. 1726 to 2010. The maps represent a time interval that integrates over decades-long periods between the dates **(D)** Water column nitrate collected monthly by the volunteer monitoring program and averaged from 1990 to 2010.

Using the composite core chronology combining all seven cores (Figure 2), we estimated the age of binned sediments used to produce isoscape and stoichioscape maps at six time periods (ca. 1726 to 2010) (Table S6). The maps were created with input data from the 85 sediment core locations (Table S7). Elkhorn Slough nitrogen isoscape maps reveal spatiotemporal shifts that suggest alterations in slough-wide nutrient availability. Significant increases through time are apparent in sediment δ^15^N values, with low values apparent through the late 1800s, and dramatic increases after the 1950s (Figure 4A). Historical isoscape maps (pre-1900) show a head to mouth gradient in nitrogen isotope values with high values near the mouth (~6‰) and lower values near the head (0-1‰). Recent isoscape maps however reveal that the δ^15^N isotopic gradient has shifted towards high δ^15^N values at both the mouth and head, and low values in the mid-estuary.

These patterns agree with known water quality gradients. Elkhorn Slough’s major tributaries (Old Salinas River Channel and Carneros Creek) – both of which convey agricultural drainage water derived from row crops (Los Huertos et al., 2001) – enter near its mouth and head, respectively. Based on water monitoring data shown in the 1990 to 2010 NO_3_^−^ map (Figure 4D), the locations at the head and mouth of the estuary, which showed high δ^15^N during the last century, are associated with the highest nitrate concentrations within the estuary. The largest sediment δ^15^N values were observed at the Harbor where water column NO_3_^−^ concentrations in excess of 1000 μM are common (Johnson et al., 2007). While NO_3_^−^ values in the upper Slough do not approach those in the lower Slough, the upper Slough is not well flushed, providing extended opportunities for nitrogen processing and thus high water column δ^15^N values.

Carbon isoscape and C/N stoichioscape maps similarly reveal spatiotemporal patterns with regions of significant change at the head and mouth of the estuary. Based on values for all 85 coring sites (mean ± standard deviation), in the 19^th^ century δ^13^C values were −24.6 ± 1.4‰, and C/N ratios were 11.9 ± 2.4 and in the most recent isoscape (ca. 1981-2010) they were −25.0 ± 1.7% and 11.2 ± 2.2‰ respectively. These values indicate that preserved estuarine organic matter clearly represented a mixture of terrestrial and aquatic organic matter sources; most samples likely consisting of a mixture of *Salicornia* (the dominant coastal wetland plant) and phytoplankton. Based on mean values there were no overall directional shifts through time, however location-specific changes were observed. Isoscape maps of historical periods (pre-1900) show uniformity of δ^13^C throughout the Slough, with a slight gradient of lighter C in the mid-upper Slough (Figure 4B). A rough head to mouth gradient was also recognized in historical C/N stoichioscape maps (pre-1900), with low C/N found near the estuary’s mouth and high C/N found near its head (Figure 4C). In the mid estuary to the head, δ^13^C values increased from the oldest isoscape until the mid-1900s. The most recent (ca. 1981-2010) δ^13^C isoscape map shows a pronounced shift to more ‘terrestrial’ signatures in the upper and lower portions of the estuary and more aquatic signatures in the mid part of the estuary.

### 3.3 Temporal analysis of isotopes and stoichiometry

**FIGURE 5:**
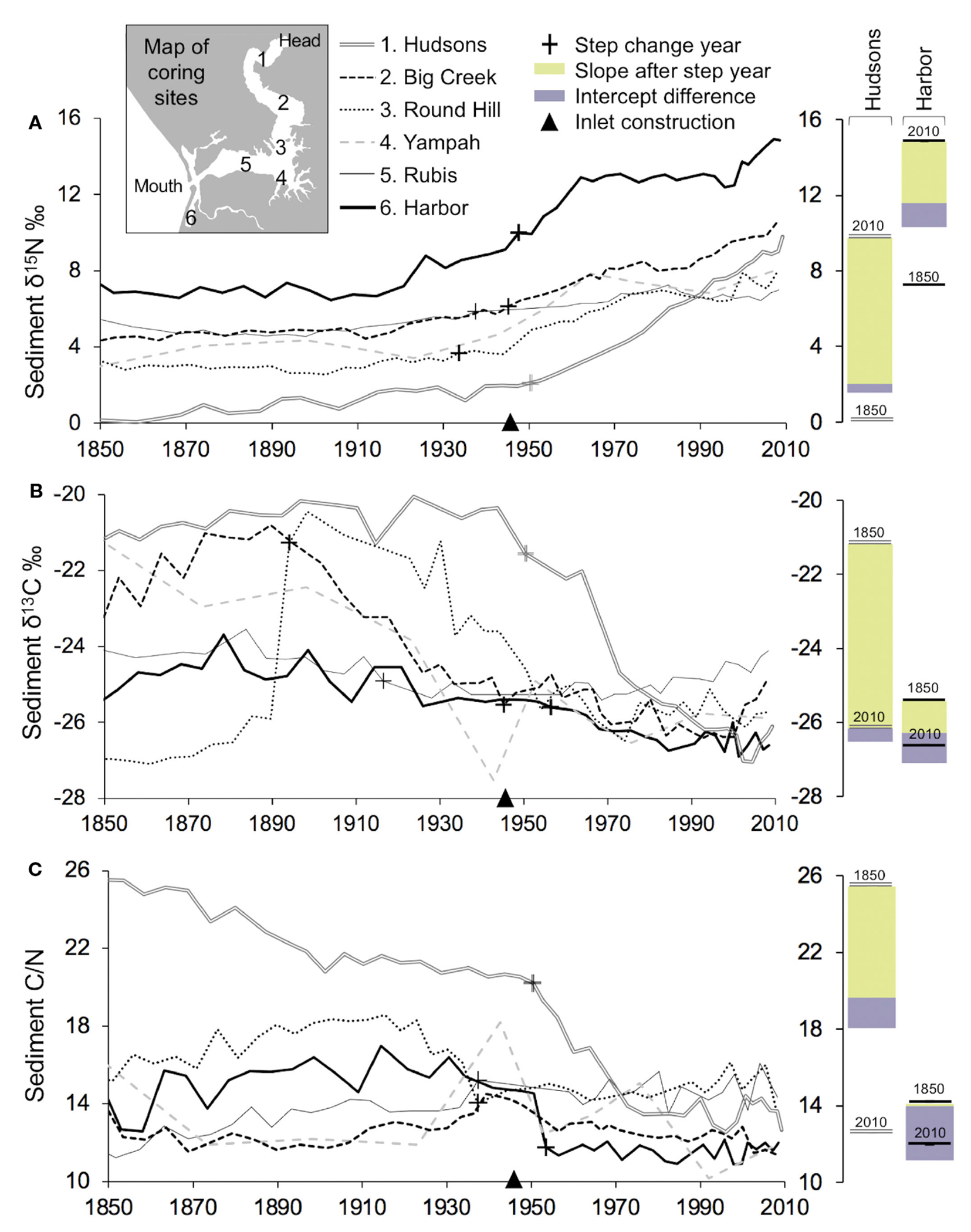
Continuous records of isotopic values and nutrient stoichiometry over 160 years at six locations in Elkhorn Slough **(A)** Sediment stable nitrogen isotopes; **(B)** stable carbon isotopes; and **(C)** the carbon to nitrogen ratio. Barplots show the change associated with the slope and intercept changes after the step change year identified statistically using a Pettitt Test (*p*-values in Figure S1).

Comparison of high-resolution isotope ratios of six dated sediment cores from 1850-2010 (Table S8) revealed baseline shifts and absolute values associated the timing of two events; (1) increasing watershed N inputs; and (2) increased marine exchange. We present the 2010 absolute δ^15^N values (ABS) and isotopic differential between the 2010 δ^15^N value minus the 1850 δ^15^N value (DIF). The ABS and DIF values were highest at the mouth of the Slough (DIF = +9.6‰ ABS = 14.8‰ for Harbor) and at its head (DIF = +8.8‰; ABS = 9.7‰ for Hudsons Landing; DIF = +7.1‰; ABS = 10.4‰ for Big Creek) (Figure 5A). The greatest changes in sediment δ^15^N values, both in terms of isotopic differential and absolute magnitude, were observed at the head and harbor in proximity to watershed N inputs. In contrast, isotopic differential and absolute values were found to be lower mid-Slough (DIF = +4.1‰; ABS = 6.9‰ for Round Hill; DIF = +4.7‰; ABS = 8.0‰ for Yampah; DIF = +2.4‰; ABS = 7.9‰ for Rubis), consistent with more marine input in the mid-estuary. In addition, C isotopic and C/N timeseries for individual sediment cores indicated changes in nutrient processing. Values pre-1900 tend to have a wider range among the six sites located across the spatial gradient of the estuary, but tend to be more tightly clustered for 2010, suggesting that a homogenization of the estuary has occurred (Figures 5B, C).

Since the mid-1900s, high resolution sedimentary analysis showed increasing δ^15^N, declining δ^13^C and C/N values, and homogenization of δ ^13^C and C/N values across sites. We investigated the alignment of the isotopic and stoichiometric shifts with the timing of (1) harbor inlet construction and increased tidal input in 1946; and (2) increasing watershed N inputs in 1940-1980. Supporting the statistical significance of these two events, timeseries analysis identified both offsets and ongoing trends in the isotopic and stoichiometric signatures at most of the coring sites (*p*-values in Figure S1). A fraction of the change of δ^15^N, δ^13^C, and C/N can be attributed to a step change point around the 1940s when the harbor was constructed (Figure 5 bar plots). However, a larger portion of the change of δ^15^N, particularity at the Hudsons and Harbor sites, is associated with a gradual trend since the 1950s, indicative of increasing N application in the watershed (Figure 5A bar plots).

Comparison of sediment stable nitrogen isotopes to water column nutrient concentrations measured by the long-term volunteer monitoring program suggested a relationship between mean water column NO_3_^−^ levels and sediment δ^15^N. Water column NO_3_^−^ concentrations (maximum annual mean = 1432 μM) were highest near the mouth of the estuary (Harbor coring site) associated with the greatest measured sediment δ^15^N values (Figure S3A). Relatively less but still high NO_3_^−^ concentrations (maximum annual mean = 642 μM) and high sediment δ^15^N values were also found at the head of the estuary (Hudsons coring site). Generally, low concentrations of nutrients and lower sediment δ^15^N values were found in the mid-channel of Elkhorn Slough, which is well-flushed. We observed heavier δ^15^N at the Harbor site, which also reports lower and more variable salinity (Figure S3C).

### 3.4 Historical nitrogen sources

**FIGURE 6:**
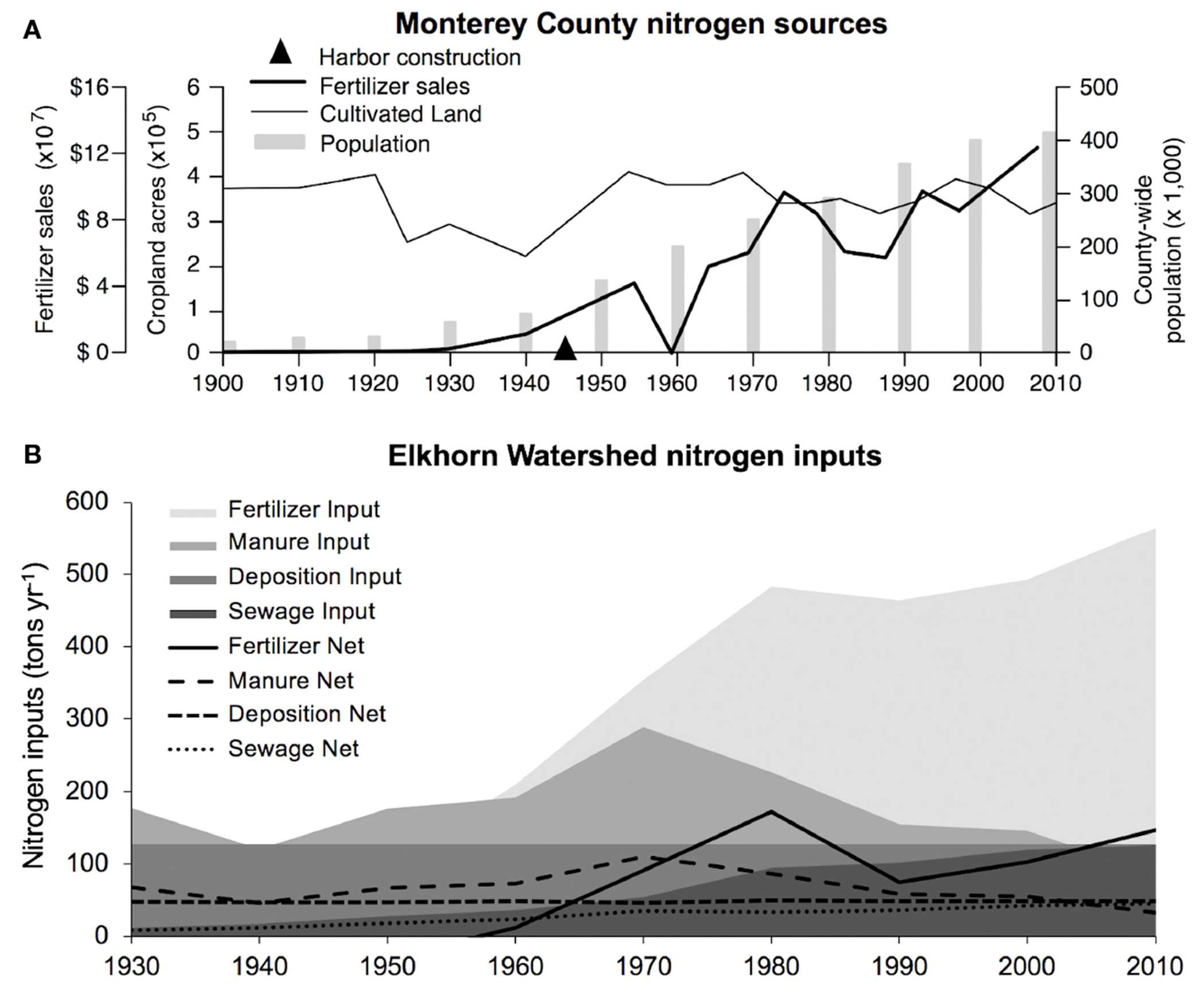
**(A)** Changes in land use and demographic data in Monterey County (fertilizer sales reported in 2016 dollars). County data accounts for indirect runoff from the larger Gabilan/Tembladero and Salinas Watersheds to the south, which intermittently contribute to Elkhorn Slough through the Old Salinas River, and are described in a prior TMDL (Osmolovsky et al., 2013). **(B)** Major source of nitrogen inputs over time estimated using the NLM tool parameterized for the Elkhorn Watershed.

Based on the NLM adapted for Elkhorn Slough in this study, the trend of nitrogen inputs in the Elkhorn Slough direct-drainage watershed and Monterey County is dominated by increasing agricultural fertilizer input (Figure 6B), with a rapidly increasing trend starting in 1940, consistent with development of synthetic fertilizer using the Haber-Bosch Process (Nixon, 1995). Changes from several sources contributed to more constant nitrogen inputs observed after 1980, including concurrent leveling off of fertilizer addition rates, higher crop production and export, and decreasing livestock numbers through reduction of herds of mostly non-dairy cattle in the Elkhorn watershed (Table S3A). Wastewater nitrogen contributes a small and slightly increasing portion of nitrogen sources in the watershed, although county-wide populations increased at a consistent rate since the 1920s (Figure 6A). This nitrogen load model parameterized for Elkhorn watershed highlights 1940-1980 as the period of rapid increases of N inputs to the direct-drainage watershed during the past century.

## 4 Discussion

### 4.1 Magnitude of nitrogen inputs over time

The multi-centennial perspective provided by sediment stable nitrogen isotopes reveled substantial increases of nitrogen levels in Elkhorn Slough. We demonstrated that modern sediment δ^15^N reproduced spatial patterns of nitrate concentration measured by a volunteer monitoring program in Elkhorn Slough (Figure 4D), and annual water column nitrate concentration corresponds with high sediment δ^15^N values (Figure S3A). Therefore, the trends of δ^15^N in core timeseries are interpreted as increasing N levels in this system over time. An incredibly strong contrast exists between current δ^15^N in Elkhorn Slough and the recent past. Modern sediment δ^15^N is higher than pre-1900 baseline values at all sites, but especially at the mouth (DIF = +9.6‰) and head (DIF = +8.8‰) of the estuary (Figure 5). These shifts over the past century at the head and mouth of Elkhorn Slough are extreme even in comparison with previously studied eutrophic estuaries during the same time period. Previous work in eutrophic estuaries reported shifts of as much as 7‰ in marsh sediments in Jamaica Bay, New York City between 1850 and 2010 (Wigand et al., 2014), and increases of up to 4‰ of δ^15^N in oysters and hard clams from Narragansett Bay and Chesapeake Bay (Oczkowski et al., 2016; Black et al., 2017). Estuaries with lower levels of nutrient pollution have supported shifts of 2-3‰ in sediment stable isotopes relative to pre-industrial times (Soto-Jiménez et al., 2003; Bender et al., 2015; Reeves et al., 2015; Velinsky et al., 2017). During the last century, the baseline shifts of N isotopic values in the mid-estuary of Elkhorn Slough were similar to other eutrophic estuaries, but greater shifts occurred at the head and mouth, indicating a considerable change in the magnitude of N inputs.

Changes of sedimentary δ^15^N in Elkhorn Slough suggest that N inputs have increased by several orders of magnitude since 1900, which was supported by our geospatial analysis of watershed inputs using a NLM adapted for Elkhorn Watershed. At the mouth of the estuary, high N loads from intensive agriculture enter the Old Salinas River channel near the Harbor coring site (Osmolovsky et al., 2013). Our analysis of the historical changes in population, land use patterns, and fertilizer application rates in the direct-drainage from Elkhorn watershed using the NLM model indicated that higher N at the head of the estuary was also impacted by increased fertilizer application, the largest contribution to N inputs since 1970 (Figure 6B). Although we did not quantify the form of fertilizer applied, ammonium vs. nitrate types can influence transport and biogeochemical processing (Subbarao and Searchinger, 2021), so further analysis of fertilizer type over time could provide additional perspective. Although population in the watershed also increased over the 20^th^ century, the population density is small enough to contribute minimally to N inputs. Historical changes in land use patterns in the Elkhorn watershed suggest slight increases in impervious surface cover due to construction of housing and paved roads, and historical decreases in cultivated lands since the 1980s, as a significant portion of the watershed is now preserved as natural landcover (Table S1). Because the areal extent of cultivated lands has declined, the high fertilizer application rate relative to N removed as crops appears to be driving the N inputs to the estuary. Atmospheric deposition plays a relatively smaller role in overall N inputs, in contrast with many Northeastern U.S. estuaries (Latimer and Charpentier, 2010; Kinney and Valiela, 2011).

### 4.2 Nutrient sources revealed by spatial gradients

**FIGURE 7:**
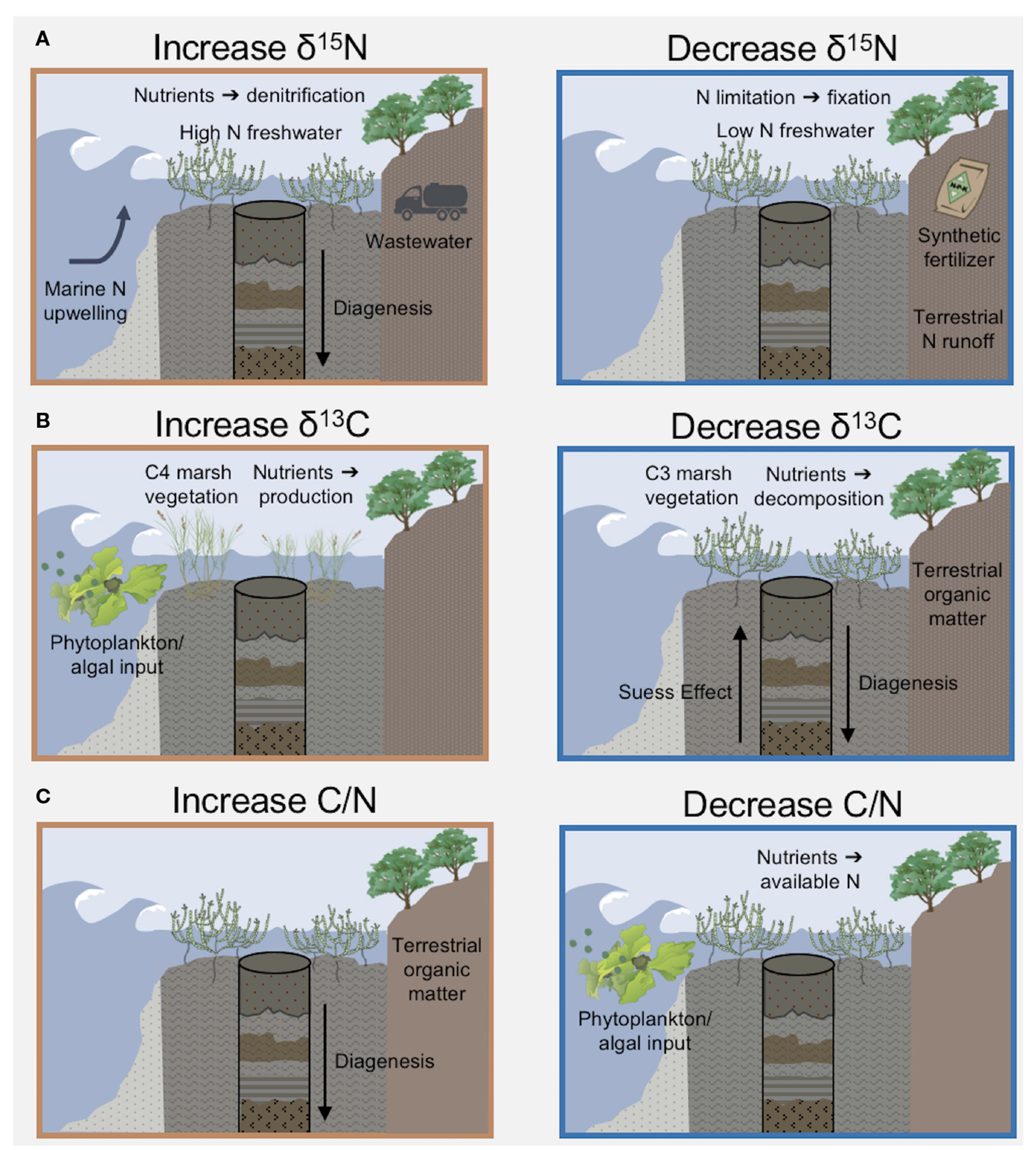
Enumeration of mechanisms, including nutrient quantity and sources, that contribute to sediment stoichiometry and stable isotope s. **(A)** N isotopes are influenced by nutrient source; NO_3_^−^ from upwelling of deep ocean water (~9‰) is typically heavier than terrestrial runoff. N in synthetic fertilizer (δ^15^N~0‰) has a lighter isotopic signature compared to wastewater (δ^15^N~10‰). High N loads increase isotopes through dentification, or low N concentrations deplete isotopes by increasing N_2_ gas fixation. **(B)** C isotopes are indicative of organic matter source from macroalgae and phytoplankton vs. terrestrial vegetation including marsh plants. Over time, the Suess Effect depleted C isotopes in the atmosphere and ocean. In sediment cores, diagenesis can cause lighter C isotopes over depth. **(C)** The C/N stoichiometric ratio is influenced by inputs of marine vs. terrestrial organic matter, because aquatic vegetation has greater N relative to C. Increased anthropogenic N loading will decrease the C/N ratio. Diagenesis increases C/N over depth in sediments. Image icons from IAN (citations Table S9).

In addition to significant changes in the magnitude of nitrogen inputs to Elkhorn Slough over the past century, a change of nutrient source was indicated by the spatial isotopic gradient. In isoscape maps prior to 1900, higher δ^15^N values (δ^15^N = 6‰) were found near the mouth of Elkhorn Slough, which is interpreted as the historical marine endmember based on proximity to the inlet, with a linear gradient towards lower δ^15^N ratios (δ^15^N = 0‰) found near the head of the Slough, indicative of the historical terrestrial endmember (Figure 7). N isotope ratios are typically higher in the marine environment, and this is especially true in central and southern California, where nitrogen advected from the Eastern Tropical North Pacific (ETNP) oxygen minimum zone near the southern tip of Baja California is heavy (δ^15^N = 10.4‰ to 14.3‰) due to high rates of marine denitrification (Voss et al., 2001; Sigman et al., 2005; White et al., 2013). This high δ^15^N water is entrained in the California Current System and upwelled along the California coast (Vokhshoori and McCarthy, 2014), and advected into Elkhorn Slough through the inlet. In contrast, low historical δ^15^N values found in the upper estuary are reflective of atmospheric N_2_ fixation by terrestrial vegetation (Santi et al., 2013). The historical (pre-1900) marine-terrestrial isotopic gradient of Elkhorn Slough was similar to that reported previously for modern pristine, upwelling-dominated estuaries where oceanic nitrate is a major N source (Huntington and Boyer, 2008; Carriquiry et al., 2016).

Recent isoscape maps (ca. 1981-2010) of Elkhorn Slough show a novel spatial pattern of nitrogen. Contrasted with the historical marine-terrestrial gradient, the modern isoscape exhibits higher δ^15^N values at both the mouth and head of the estuary. Timeseries analysis of high-resolution sediment δ^15^N indicated that a fraction of this change can be attributed to increased exchange of marine water through to inlet to Monterey Bay. Modern studies show that Monterey Bay water entering through the inlet is a dominate source of water to the lower estuary (Chapin et al., 2004; Johnson et al., 2007). Monterey Bay deep water column nitrate water has an average δ^15^N signature of +9.0‰ (Wankel et al., 2009), so exchange of this marine water through the constructed inlet would have imparted a heavier δ^15^N signature compared to our values of sediment δ^15^N in the estuary prior to the opening of the inlet in 1946. However, our timeseries analysis indicates that the majority of temporal change of δ^15^N occurred as a progressive trend concurrent with increasing anthropogenic N inputs to the watershed and in proximity to terrestrial inputs at both the head and mouth, which support watershed nutrient loads as a significant source of change during the last century. Furthermore, modern δ^13^C ratios are indicative of terrestrial organic matter sources at the mouth and head of the estuary in accordance with increased watershed inputs.

In the modern estuary, terrestrial runoff with high N concentrations from agriculture imparts a heavier δ^15^N signature than the marine end-member and enters at the head and mouth of the estuary. The Old Salinas River (OSR) channel, the location of our Harbor coring site near the mouth, has a water column δ^15^N nitrate value of +14.5‰ ± 2.9‰ (Wankel et al., 2009). The channel conveys terrestrial runoff with low salinities from the Moro Cojo, Gabilan/Tembladero, and Salinas Watersheds. Fresher OSR runoff enters close to the marine inlet complicating the salinity gradient of this estuary. Although the water column δ^15^N for Carneros Creek (CC), the location of our Hudsons coring site, is previously unmeasured, our results showing sediment high δ^15^N at the head of the estuary demonstrate that high levels of denitrified N are also imparted by this freshwater input. An inverse relationship of δ^15^N with salinity is observed in eutrophic estuaries if the freshwater end-member contributes high N levels, for example, δ^15^N measured in *Potamocorbula amurensis* clams from San Francisco Bay (North Bay) (Fry, 2002). Although synthetic fertilizer imparts a light signature (δ^15^N ~0%), high N loads in agricultural runoff will rapidly increase the N isotopic signature through denitrification and biotic uptake during transport in streams (Kellman and Hillaire-Marcel, 2003). Our observation of the development of a modern spatial pattern with high δ^15^N in the terrestrial, fresher end-members of OSR at the mouth and CC at the head of the estuary underscores the contribution of anthropogenic N loads.

The uneven spatial distribution of stable N isotopes is indicative of marine exchange as another driver of the patterns of nutrient processing. In the middle portion of Elkhorn Slough, sediment stable N isotopes differ from the signatures of eutrophication at the head and mouth of the estuary. We attribute lower δ^15^N values in the mid-slough to both their relative distance from direct sources of agricultural runoff and increased marine exchange (Wasson et al., 2017). The δ^15^N values in the mid-estuary were similar to those reported elsewhere on the California coast (Fong and Zedler, 2000; Cloern et al., 2002) and have δ^15^N signatures that reflect less nutrient enrichment than many southern California estuaries (Cohen and Fong, 2006). Similarly, other water quality indicators assessed previously at Elkhorn Slough, such as hypoxia and sediment quality also point to water quality in mid-Slough as being less impaired than the upper Slough (Hughes et al., 2011). Furthermore, between the 1900s and the present, the carbon isotope and C/N stoichiometric ratios appeared to decrease in overall range among the six coring sites. We interpret this homogenization of the sites relative to the baseline range as evidence of an increased marine input associated with increased tidal exchange from the opening of the constructed inlet in 1946 (Woolfolk, unpublished data).

### 4.3 Potential of historical isoscapes for regulatory policy

As described in the previous sections, our findings support the use of sediment stable nitrogen isotopes as a multi-centennial proxy indicative of the magnitude and source of N in estuaries. Identifying changing N levels in coastal estuaries is significant because it can help establish a historical baseline to compare to the modern system (Duarte et al., 2009). Timeseries of δ^15^N are increasingly used to identify baseline shifts in N load based on analysis of modern compared to historical sediments and organisms. For example, sediment δ^15^N data identified changes of N inputs associated with the onset of wastewater treatment in Jamaica Bay, NY (Wigand et al., 2014). Additionally, bivalve shell δ^15^N data reflected water quality improvements following local wastewater management in Narragansett Bay, RI (Oczkowski et al., 2016). Further, oyster shell δ^15^N data constrained the timing of increased sediment N loads associated with anthropogenic activity in the Chesapeake Bay Watershed (Black et al., 2017). Prior baseline studies often provide isotope timeline data with separation of locations over time, but isoscapes with high-spatial resolution are a recent application to historical and palaeo-contexts (Reade et al., 2023).

The use of isoscapes to detect relative shifts in N dynamics, long before the establishment of water quality monitoring, has important applications for N regulation policy. Elkhorn Slough’s water quality has been extremely well-measured using a combination of *in-situ* analyzers and monthly sampling, which provide important perspectives about nitrogen dynamics over the past 30 years. Two decades of data demonstrated that nutrient availability is associated with tidal exchange and seasonal runoff (Caffrey et al., 2007). High-resolution temporal data revealed that tidal cycles transport marine nitrate through the inlet (Chapin et al., 2004), and pulses of nitrate from runoff are propagated by tides (Johnson et al., 2007). Additionally, spatial patterns showed that expression of eutrophication is enhanced behind tidal restrictions (Hughes et al., 2011). While these studies raised concerns about symptoms of eutrophication, biological assimilation can mask the detection of elevated N inputs by monitoring programs (Watson et al., 2017; Jeppesen et al., 2018; Sánchez et al., 2023). Datasets of multiple monitoring parameters including dissolved and sediment nutrients, oxygen and pH, phytoplankton and microalgal abundance each provide useful information for managers when setting goals for N reduction but are limited by their 30-year timescale. Approximately 65-80% of the change of sediment δ^15^N between 1850 and 2010 occurred prior to water quality measurements in 1988 (Figure 5A). Therefore, the onset of monitoring does not represent a reference state for this system. We posit that Elkhorn Slough isoscapes pre-1885 represent a quantitative baseline to compare modern N dynamics and spatial patterns. Using historical isoscapes and baseline data, Table 1 outlines specific policy recommendations for this system, as an example for how evidence provided by isoscapes can be applied to guide local N regulation policy.

### 4.4 Limitations and complexities of isotopes

Isotopic signatures are influenced by many estuarine processes in addition to anthropogenic nutrient inputs (Figure 7). In Elkhorn Slough, the correlation of sediment δ^15^N with available water column nitrate concentrations (Figure S3A) was not strong enough to use linear regression of sediment δ^15^N to model historical nitrate concentrations or suggest quantitative nitrate targets based on δ^15^N data alone. Denitrification during N transport and within the estuary both contribute to the association of higher nutrient levels with higher δ^15^N signatures. The δ^15^N values cannot distinguish where denitrification is occurring, whether during N transport from the watershed or local processing within the estuary water column and sediment. Furthermore, denitrification is an anaerobic process, so hypoxia of the water column associated with eutrophication and hypoxic sediments increase δ^15^N values (Ganeshram et al., 2000; Sánchez et al., 2022). Significant fractionation also occurs during nitrate uptake by biological assimilation in the estuary, therefore incomplete nitrate utilization would result in isotopically lighter δ^15^N in organic matter that is then incorporated into sediments (Altabet, 2001). Therefore, in areas of the modern estuary with high nitrate concentrations, incomplete nitrate utilization may drive a water column δ^15^N signature that is even higher than represented in the sediments (White et al., 2013). Finally, degradation of N within the sediment layers over time increases δ^15^N deeper in the core (Sánchez et al., 2022). We observed a trend of increasing δ^15^N in modern sediments, opposite of the trend imparted by diagenesis, so the change may be even more pronounced than observed in sediment timeseries.

Sediment δ^13^C and C/N ratios are also impacted by competing mechanisms in estuaries (Figure 7), which may influence the changes in the δ^13^C and C/N timeseries (Figures 5B, C). For example, shifts between vegetation using C3 and C4 photosynthetic pathways may alter sediment δ^13^C values, therefore dominant marsh vegetation species may influence δ^13^C values and the patterns observed before 1900 (Byrne et al., 2001). Another factor is that nitrogen loading may reduce C/N ratios, as N is incorporated in tissues as a function of supply in nutrient limited environments (U.S. EPA, 2002). Opposing the lighter C isotope signal of terrestrial inputs, aquatic ecosystems often shift to heavier δ^13^C in association with eutrophic conditions in accordance with partial carbon dioxide limitation (Voβ and Struck, 1997). Over time, diagenesis increases C/N ratios and lightens δ^13^C values (Galman et al., 2009; Brahney et al., 2014). Lastly, the Suess effect, shorthand for lighter atmospheric δ^13^C from fossil fuel combustion, can cause a shift by about −1.5‰ of δ^13^C since 1850 (Verburg, 2007).

## 5 Conclusions

This study demonstrated the ability of whole-estuary isoscape and stoichioscape maps to provide a historical perspective on nutrient levels and sources in a eutrophic estuary. Stable N isotope analysis revealed baseline shifts in δ^15^N values, symptomatic of increased N inputs. The shifts in sediment δ^15^N values in this study are greater than those reported by previous historical and gradient studies (Wigand et al., 2014; Oczkowski et al., 2016; Watson et al., 2018) and suggest that N availability increased by orders of magnitude relative to the 19^th^ century, supported by geospatial analysis of historical N inputs to the watershed. Additionally, we report novel patterns of the marine-terrestrial nutrient gradient driven by changing sources and increased tidal inputs which are revealed by the high spatial resolution of our mapping approach. This study supports the use of historical isoscapes, alongside other datasets, as a robust tool to identify evidence of nitrogen pollution over century-long timescales to guide nutrient reduction goals in sensitive coastal areas.

## Supplementary Material

Supplement1

## Figures and Tables

**TABLE 1:** Examples of local policy recommendations for Elkhorn Slough estuary using the isoscape maps and supporting historical data from this study.

Policy recommendations	Evidence from this study
(1) Reduce nitrogen loads to the estuary	Our timeseries data show much higher modern nitrogen levels compared to baseline levels, and greater change from baseline than many other impacted estuaries (Figure 5A).
(2) Focus on reducing fertilizer use in the watershed	Our model of nitrogen loads during the past century suggests that fertilizer application is key driver of change over time (Figure 6B).
(3) Reduce loads entering at the mouth and head of estuary	Maps indicate separate and significant sources in each area (the former related to the OSR input and the latter to upper Elkhorn watershed) (Figure 4A).
(4) Avoid long residence times within the estuary	The relationship of isotopes with water quality monitoring data suggests that both nutrient concentrations and residence time affect impairment (Figure S3A).

## Data Availability

The original data generated in the study is uploaded to a publicly accessible online database Dryad [https://doi.org/10.5061/dryad.3ffbg79q6]. In addition, publicly available water quality monitoring datasets were analyzed in this study and information about accessing these data can be found here: [https://www.elkhornslough.org/research-program/waterquality-weather-monitoring/volunteer-monitoring/].
