# Supplementary material for: Use of historical isoscapes to develop an estuarine nutrient baseline
Source: Front Mar Sci. Author manuscript; Available in PMC 2024 Sep 6. (PMC10563801; doi:10.3389/fmars.2023.1257015)
Supplement: Supplement1 [file NIHMS1934301-supplement-Supplement1.docx]

Supplementary Material

Use of historical isoscapes to develop an estuarine nutrient baseline

Lena K. Champlin^1^*, Andrea Woolfolk^2^, Autumn J. Oczkowski^3^, Audrey Rittenhouse^1^, Andrew B. Gray^4^, Kerstin Wasson^2,5^, Farzana I. Rahman^1,6^, Paula Zelanko^1^, Nadine B. Quintana Krupinski^7,8^, Rikke Jeppesen^2^, John Haskins^2^, and Elizabeth B. Watson^9^

*** Correspondence:**

Lena K. Champlin

[lenakchamplin@gmail.com](mailto:lena_champlin@alumni.brown.edu)

**Contents**

This document includes the supplementary Figures (Figures S1 to S3) and Tables (Tables S1 to S9) referenced in the paper.

# Supplementary Figures

**Supplementary Figure S1.** Timeseries trend analysis of N and C isotopes and the C/N ratio from six high resolution cores from 1850 to 2010 (Fig. 6). Change points in the timeseries were identified using the Pettitt Test, then timeseries were split “before” and “after” the year identified. Trend analysis was performed using linear regression on the split datasets, yielding the slope after the change point and the difference of the y-intercept between the before and after datasets. Blue: significant step change at the year 1946 ±10. Pink: significant trend in the split datasets. Significance codes: *** *p* ≤ 0.001; ** *p* ≤ 0.01; * *p* ≤ 0.05; ns (not significant).


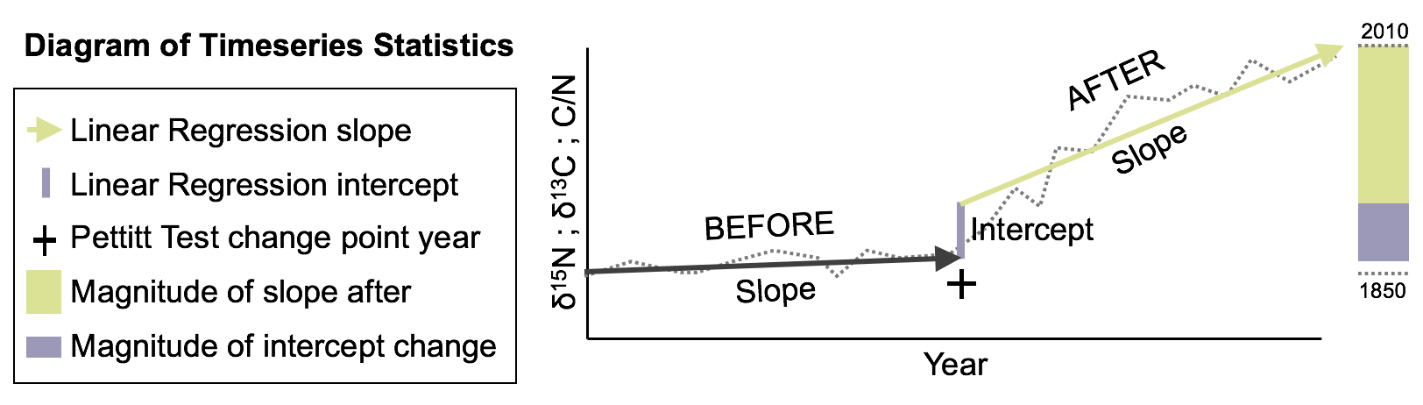


|  | **Pettitt Test** | | **Linear Regression** | | | | |
| --- | --- | --- | --- | --- | --- | --- | --- |
| **Site** | **Change point**  **Year *p*-value** | | **Before**  **Slope *p*-value** | | **After**  **Slope *p*-value** | | **Difference of Intercept** |
| **δ^15^N ‰** | | | | | | | |
| Harbor | 1948 | <0.001*** | 0.023 | <0.001*** | 0.052 | <0.001*** | 1.29 |
| Rubis | 1938 | <0.001*** | 0.004 | 0.211 ns | 0.008 | 0.098 ns | 1.53 |
| Yampah | 1953 | 0.066 ns | 0.019 | 0.080 ns | -0.0009 | 0.967 ns | 3.12 |
| Round Hill | 1934 | <0.001*** | 0.003 | 0.124 ns | 0.052 | <0.001*** | 0.37 |
| Big Creek | 1945 | <0.001*** | 0.014 | <0.001*** | 0.059 | <0.001*** | 0.71 |
| Hudsons | 1950 | <0.001*** | 0.019 | <0.001*** | 0.129 | <0.001*** | 0.48 |
| **δ^13^C ‰** | | | | | | | |
| Harbor | 1956 | <0.001*** | -0.007 | 0.019 * | -0.016 | <0.001*** | -0.81 |
| Rubis | 1917 | 0.001** | -0.008 | <0.001*** | 0.009 | <0.001*** | -5.60 |
| Yampah | 1943 | 0.086 ns | -0.056 | 0.052 ns | -0.012 | 0.469 ns | 0.64 |
| Round Hill | 1894 | 0.001** | 0.027 | <0.001*** | -0.049 | <0.001*** | 6.52 |
| Big Creek | 1945 | <0.001*** | -0.034 | <0.001*** | -0.012 | 0.0329 * | 1.11 |
| Hudsons | 1950 | <0.001*** | 0.006 | 0.012 * | -0.089 | <0.001*** | 0.35 |
| **C/N** | | | | | | | |
| Harbor | 1954 | <0.001*** | 0.001 | 0.851 ns | 0.002 | 0.729 ns | -2.94 |
| Rubis | 1938 | <0.001*** | 0.031 | <0.001*** | 0.008 | 0.466 ns | -1.47 |
| Yampah | 1992 | 0.524 ns | NA | NA | NA | NA | NA |
| Round Hill | 1937 | <0.001*** | 0.021 | 0.0103 * | 0.011 | 0.0474 * | -3.72 |
| Big Creek | 1912 | 0.139 ns | NA | NA | NA | NA | NA |
| Hudsons | 1950 | <0.001*** | -0.055 | <0.001*** | -0.097 | <0.001*** | -1.58 |

# Supplementary Figure S2. Percent carbon (%C) relative to the percent nitrogen (%N) in the focal sediment cores for the top 50 cm depth.

**
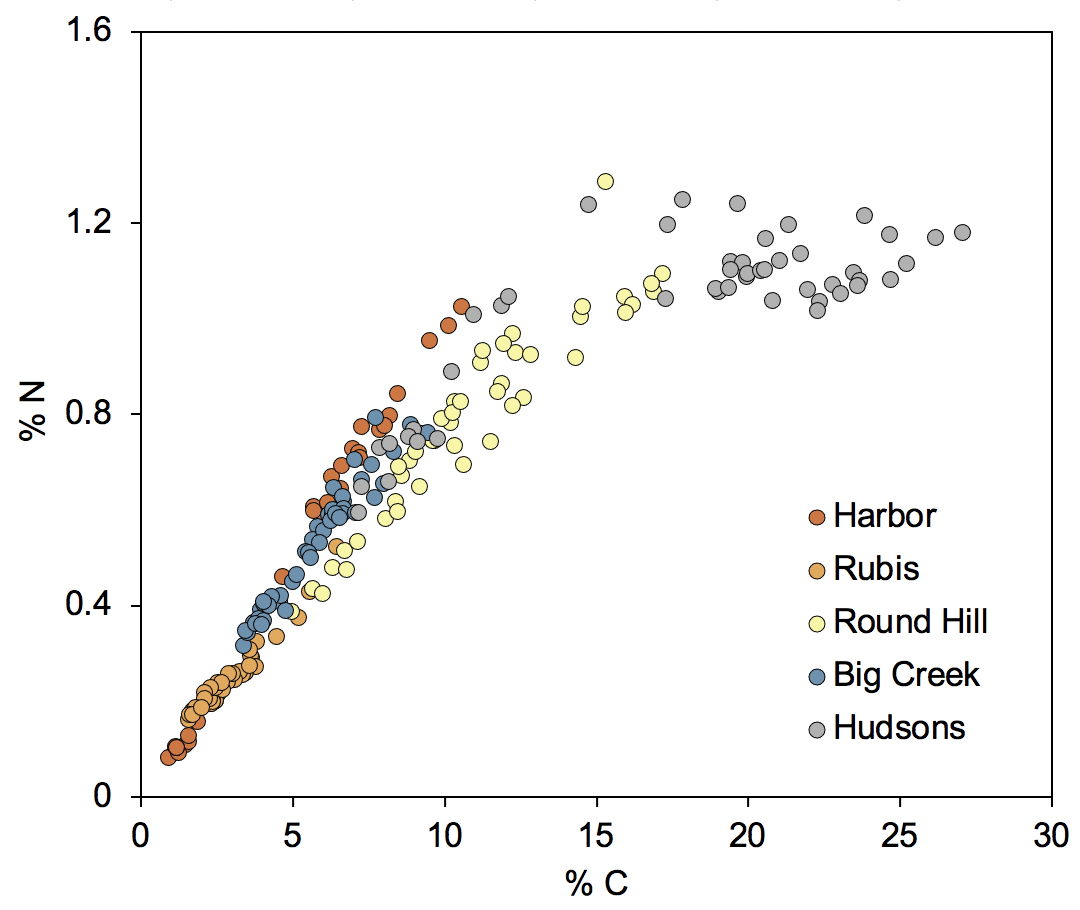
**

**Supplementary Figure S3.** Sediment N stable isotopes from three cores compared to water quality parameters including (a) Water nitrate concentrations; and (b) Water salinity levels presented as annual averages from 1990 to 2010. Water column chemistry data were collected monthly by the volunteer monitoring program at three sites that correspond to coring sites: Harbor = Portero Road North (PRN), Hudsons = Hudsons Landing West (HLW), Big Creek = Kirby Park (KP). Surface sediment N isotopes collected at 20 sites during the summer of 2016 compared to (c) Water nitrate concentrations; and (d) Water salinity levels shown as the monitoring site mean during 2015 to 2016.

**
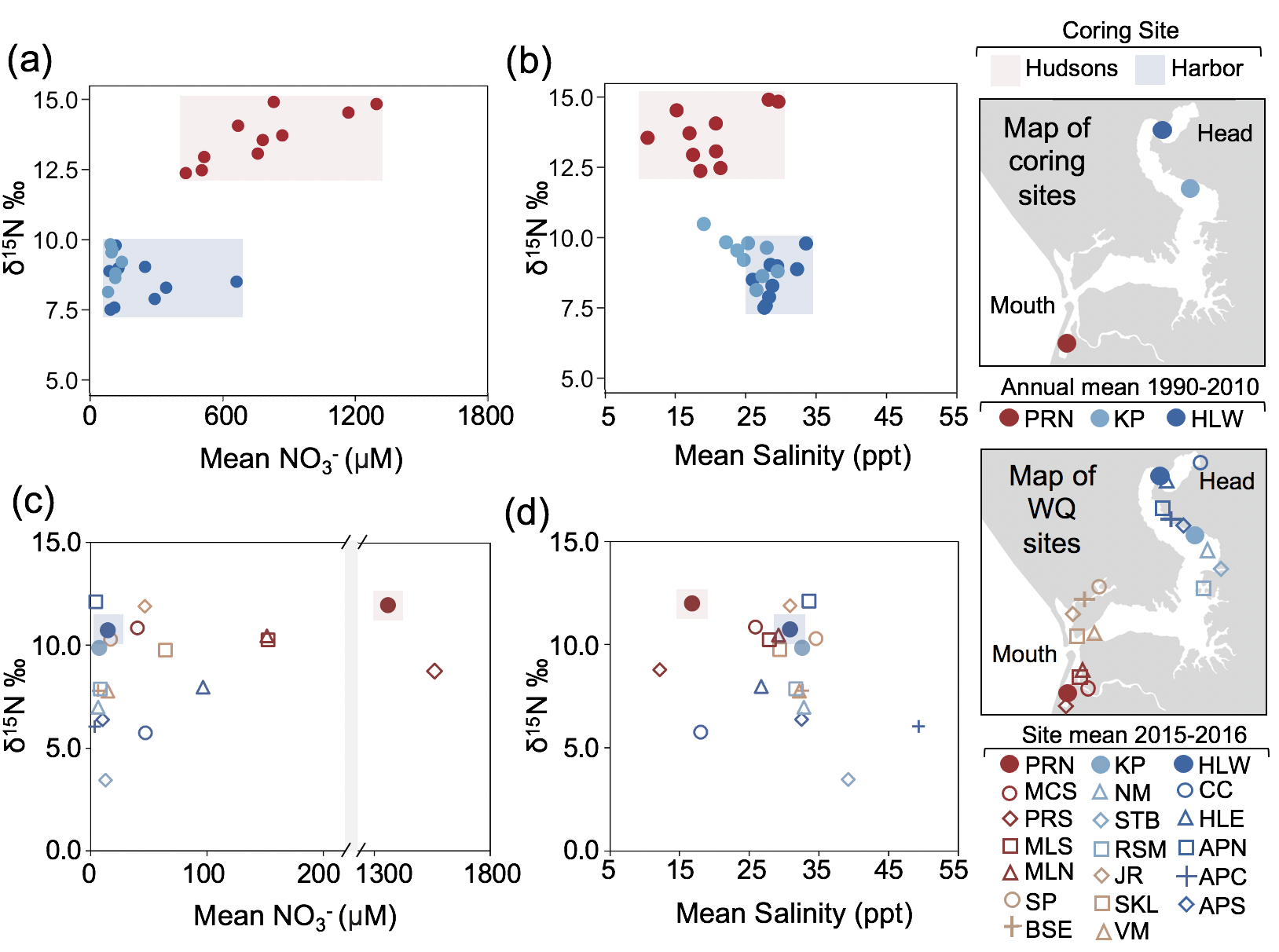
**

# Supplementary Tables

**Supplementary Table S1.** Parameters used for the nitrogen loading model each decade from 1930 to 2010. This adaptation of the model includes nitrogen inputs and initial attenuation in land cover, but it excludes transportation estimates in the groundwater to the estuary. Parameters highlighted in blue were calculated specifically for the study site and parameters in yellow were added to the model to incorporate local processes.

|  | **1930** | **1940** | **1950** | **1960** | **1970** | **1980** | **1990** | **2000** | **2010** |
| --- | --- | --- | --- | --- | --- | --- | --- | --- | --- |
| **GENERAL PARAMETERS** |  |  |  |  |  |  |  |  |  |
| Agricultural fertilization (kg N ha^-1^ y^-1^) | 0.6 | 1.7 | 5.3 | 11.6 | 20.0 | 27.0 | 25.8 | 27.3 | 31.5 |
| Livestock manure^1^ (kg N ha^-1^ y^-1^) | 10.5 | 7.1 | 10.4 | 11.3 | 17.0 | 13.4 | 9.1 | 8.6 | 5.0 |
| Exported N in crops^2^ (kg N ha^-1^ y^-1^) | 3.5 | 5.5 | 5.3 | 6.8 | 7.4 | 7.2 | 12.3 | 11.7 | 11.7 |
| Atmospheric deposition (kg N ha^-1^ y^-1^) | 7.5 | 7.5 | 7.5 | 7.5 | 7.5 | 7.5 | 7.5 | 7.5 | 7.5 |
| Golf/ Lawn fertilization (kg N ha^-1^ y^-1^) | 0.4 | 0.5 | 0.5 | 0.7 | 0.9 | 1.5 | 1.6 | 1.7 | 1.8 |
| Houses (count) | 240 | Int | 661 | Int | 2462 | 5200 | 5687 | 6343 | 6893 |
| Houses with cesspools^3^ (%) | 100 | 100 | 100 | 100 | 100 | 10.6 | 10.6 | 10.6 | 10.6 |
| Houses with public sewers^3^ (%) | 0.0 | 0.0 | 0.0 | 0.0 | 0.0 | 15.0 | 15.0 | 15.0 | 15.0 |
| Houses with septic^3^ (%) | 0.0 | 0.0 | 0.0 | 0.0 | 0.0 | 72.4 | 72.4 | 72.4 | 72.4 |
| Population (count) | 2298 | 3315 | 5251 | 6774 | 10156 | 17775 | 19214 | 22490 | 23660 |
| Per capita N input (kg N person^-1^ yr^-1^) | 4.82 | 4.82 | 4.82 | 4.82 | 4.82 | 4.82 | 4.82 | 4.82 | 4.82 |
| **LAND COVER** |  |  |  |  |  |  |  |  |  |
| Total watershed area (ha) | 15390 | 15390 | 15390 | 15390 | 15390 | 15390 | 15390 | 15390 | 15390 |
| Forested natural lands (ha) | 1790 | 2234 | 1820 | 1773 | 1806 | 2703 | 2100 | 3270 | 3272 |
| Urban impervious (ha) | 65 | 73 | 109 | 364 | 187 | 540 | 346 | 605 | 626 |
| Agricultural area (ha) | 1222 | 1130 | 1754 | 1970 | 2341 | 2727 | 1441 | 1702 | 1823 |
| Wetlands (ha) | 1683 | 1146 | 1089 | 780 | 802 | 1036 | 724 | 1418 | 1426 |
| Low vegetation and open lands (ha) | 10446 | 10495 | 10209 | 10049 | 9310 | 7761 | 10167 | 7348 | 7144 |
| Golf courses and lawns^4^ (ha) | 68 | 84 | 100 | 168 | 236 | 443 | 480 | 529 | 571 |
| **ATTENUATION COEFFICIENTS** |  |  |  |  |  |  |  |  |  |
| Retention in natural vegetation | 0.65 | 0.65 | 0.65 | 0.65 | 0.65 | 0.65 | 0.65 | 0.65 | 0.65 |
| Retention in low vegetation | 0.62 | 0.62 | 0.62 | 0.62 | 0.62 | 0.62 | 0.62 | 0.62 | 0.62 |
| Retention in agricultural lands | 0.62 | 0.62 | 0.62 | 0.62 | 0.62 | 0.62 | 0.62 | 0.62 | 0.62 |
| Gaseous losses from fertilizer | 0.39 | 0.39 | 0.39 | 0.39 | 0.39 | 0.39 | 0.39 | 0.39 | 0.39 |
| Losses in septic tanks and leaching | 0.40 | 0.40 | 0.40 | 0.40 | 0.40 | 0.40 | 0.40 | 0.40 | 0.40 |
| Losses in plumes of septic and cesspools | 0.34 | 0.34 | 0.34 | 0.34 | 0.34 | 0.34 | 0.34 | 0.34 | 0.34 |

^1^Number of livestock multiplied by the type-specific N content in manure

^2^Weight of each crop produced multiplied by the type-specific N content

^3^Estimated percent of houses with each sewage treatment type based on a modern subsample

^4^Average lawn area based on a modern subsample multiplied by the number of houses, plus golf course area

Int= Values interpolated from surrounding time points

**Supplementary Table S2.** Data sources for input parameters of the nitrogen loading model.

| **Parameter** | **Temporal Range** | **Spatial Range** | **Source** |
| --- | --- | --- | --- |
| Watershed Area | Modern | Watershed | Dickert and Tuttle 1985;  ESNERR and ESF 2021 |
| Atmospheric deposition | 1945-2020^1^  Every 15 years | Georeferenced  map image^4^ | Viers et al. 2012, Appendix |
| Agricultural fertilization | 1925-2012^2^  Annual | Monterey County^3^ | California Department of Agriculture 1925-2012 |
| Livestock number | 1930, -40, -50, -59, -69,  -78, -87, -97, 2007, -12^1^ | Monterey County^3^ | United States Department of Agriculture 1850-2012 |
| Population  number | 1930, -40, -50, -60  1970, -80  1990, 2000, -10 | Division^4^  Tract^4^  Block^4^ | Census of the US 1930-1960;  Census of the US 1930-1960;  **NHGIS Manson et al. 2012** |
| Number of houses | 1930, 1950^1^  1970, 1980  1990, 2000, -10 | County^4^  Tract^4^  Block^4^ | **NHGIS Manson et al. 2012** |
| Land cover area | 1931, -49, -56, -66, 71, -80^1^  1992, 2001, -11^1^ | Watershed Watershed | Dickert and Tuttle 1985;  U.S. Geological Survey (USGS) 2000-2014 |
| Sewage treatment | Modern, 1970 | Subsample of 35 houses in watershed | Real estate database  Zillow, Inc. 2021 |
| Crop type number | 1930, -40, -50, -59, -69,  -78, -87, -97, 2007, -12^1^ | Monterey County^3^ | United States Department of Agriculture 1850-2012 |
| Lawn and Golf area | Modern | Subsample of 35 houses in watershed | 2021 Aerial Imagery |

^1^To compile data with a differing intervals, the values were linearly interpolated to calculate a value on the even decade year.

^2^Data with a higher temporal resolution was averaged with the decade year as the midpoint.

^3^To convert county-level data to the watershed area, the parameter was multiplied by 0.0179, which is the fraction of the county area that the watershed covers spatially.

^4^Higher resolution spatial data was clipped the watershed area.

**Supplementary Table S3.** Coefficients used to calculate N loads in agricultural production based on literature review of N levels in (a) livestock manure and (b) types of crops.

1. **N load per livestock type**

| **Livestock (number)**  **by year^1^** | **Dairy cattle** | **Other cattle** | **Horses mules** | **Swine** | **Sheep & goats** |
| --- | --- | --- | --- | --- | --- |
| 1930 | 290 | 1077 | 132 | 263 | 828 |
| 1940 | 242 | 548 | 18 | 348 | 177 |
| 1950 | 178 | 1480 | 57 | 248 | 150 |
| 1960 | 145 | 1867 | 35 | 159 | 234 |
| 1970 | 103 | 3203 | 34 | 154 | 306 |
| 1980 | 70 | 2611 | 32 | 61 | 134 |
| 1990 | 82 | 1702 | 42 | 23 | 68 |
| 2000 | 60 | 1669 | 33 | 7 | 39 |
| 2010 | 15 | 1025 | 16 | 2 | 49 |
| N in manure  (lb/head/day) | 1.02 | 0.43 | 0.46 | 0.47 | 0.036 |
| Reference for  manure N | Chang et al. 2003* | Arogo et al. 2001* | Buchanan 2003* | Chastain et al. 2003* | Ogejo and Wildeus 2010* |

1. **N load per crop type**

| **Crop (tons) per year^2^** | **Small Fruit** | **Grapes** | **Sugar Beets** | **Hay** | **Potatoes** | **Orchard fruits** |
| --- | --- | --- | --- | --- | --- | --- |
| 1930 | 0.3 | 0.2 | 0.3 | 33 | 0.9 | 9.1 |
| 1940 | 0.2 | 0 | 36.6 | 23 | 0.1 | 5 |
| 1950 | 1.7 | 0 | 33.7 | 26 | 0.9 | 2.9 |
| 1960 | 10.4 | 0.5 | 55.1 | 28.9 | 9.6 | 3.3 |
| 1970 | 17.6 | 7.4 | 34.8 | 16.8 | 15.9 | 4.6 |
| 1980 | 31.1 | 34 | 20.9 | 16 | 6.9 | 2.6 |
| 1990 | 55.8 | 58.4 | 12.4 | 9.7 | 1.5 | 1.9 |
| 2000 | 108 | 81 | 0.4 | 7.7 | 1.5 | 1 |
| 2010 | 108 | 81 | 0 | 3.9 | 1.5 | 1 |
| N in crops (%) | 1.44 | 1.54 | 1.75 | 2.1 | 1.25 | 1.44 |
| Reference for crop N | Bishop 1930* | Boone et al. 1984* | Wagner et al. 2016* | Woyema et al. 2012* | Majic et al. 2007* | Wardynski, Isleib 2018* |
| **Crop (tons) per year*** | **Wheat** | **Barley** | **Corn** | **Nuts** | **Oats** | **Beans Peas** |
| 1930 | 3.7 | 5.3 | 0.1 | 0.1 | 0.3 | 6 |
| 1940 | 3.9 | 7.2 | 0.1 | 0.2 | 0.3 | 16.9 |
| 1950 | 5.3 | 9.4 | 0 | 0.4 | 0.2 | 9.9 |
| 1960 | 2.5 | 10.5 | 0.5 | 0.3 | 0.1 | 10.2 |
| 1970 | 2.1 | 10 | 1.2 | 0.2 | 0 | 6.2 |
| 1980 | 1.5 | 8.1 | 1 | 0.2 | 0.1 | 2.2 |
| 1990 | 1.7 | 5.3 | 0.3 | 0.1 | 0.1 | 1.2 |
| 2000 | 2.1 | 2.4 | 0.1 | 0.2 | 0.1 | 0.8 |
| 2010 | 1 | 1.1 | 0 | 0.2 | 0 | 0.3 |
| N in crops (%) | 1.8 | 2.7 | 2.5 | 0.6 | 2 | 1.5 |
| Reference for crop N | Delahunty 2015* | Dixon et al. 2019* | Jarvis-Shean 2015* | Van Eerd et al. 2012* | Sullivan et al. 2020* | Reinprecht et al. 2020* |

**Footnotes for Table S3**

^1^Weight of crops harvested were from agricultural census years (Census of the US 1930-2007; 2012), so linear interpolation estimated the number on the decade.

^2^Livestock numbers were from agricultural census years (Census of the US 1930-2007; 2012), so linear interpolation estimated the number on the decade.

*Chang A, Harter T, Letey J,… Zhang R. 2003. Managing Dairy Manure in the Central Valley of California. University of California Division of Agriculture and Natural Resources Committee of Experts on Dairy Manure Management, available at https://groundwater.ucdavis.edu/files/136450.pdf.

*Arogo J, Westerman PW, Heber AJ, Robarge WP, Classen JJ. 2001. Ammonia Emission form Animal Feeding Operations. White Paper prepared for National Center for Manure and Animal Waste Management, available at https://elibrary.asabe.org/abstract.asp?aid=4136.

*Buchanan M. 2003. Horse Manure Management: A Guide for Bay Area Horse Keepers. Bay Area Manure Management Program, available at https://www.marincounty.org/-/media/files/departments/pw/mcstoppp/residents/horse-manure-mangement.PDF.

*Chastain JP, Camberato JJ, Albrecht JE, Adams J, III. 2003. Chapter 3: Swine Manure Production and Nutrient Content. Clemson University, available at https://www.clemson.edu/extension/camm/manuals/swine/sch3a_03.pdf.

*Ogejo J, and Wildeus S. 2010. Technical Note: Estimating Goat and Sheep Manure Production and their Nutrient Contribution in the Chesapeake Bay Watershed. Applied Engineering in Agriculture 26(6):1061-1065.

*Bishope LR. 1930. The Nitrogen Content and “Quality” of Barley. Meeting of the London Section, available at https://onlinelibrary.wiley.com/doi/pdf/10.1002/j.2050-0416.1930.tb05271.x. 352-269.

*Boone LV, Vasilas BL, Welch LF. 1984. The nitrogen content of corn grain as affected by hybrid, population, and location. *Communications in Soil Science and Plant Analysis*, *15*(6), 639–650.

*Wagner M, Nichols K, Milliron BJ, Volpe S, Muniz J. 2016. Farming Systems Trial: Nutrient Analysis of Organic Versus Conventional Oats. Drexel University.

*Woyema A, Bultosa G, Taa A. 2012. Effect of Different Nitrogen Fertilizer Rates on Yield and Yield Related Traits for Seven Durum Wheat (Triticum turgidum L. var Durum) Cultivars Grown at Sinana, South Eastern Ethiopia. African Journal of Food, Agriculture, Nutrition and Development 2(3).

*Majic A, Poljak M, Sabljo A, Knezovic Z. 2007. Nitrogen nutrition impact on quantitative traits of early potato (Solanum tuberosum L.). Bulletin of University of Agricultural Sciences and Veterinary Medicine, Cluj-Napoca. Horticulture 64(1–2): 246–250.

*Wardynski F, and Isleib J. 2018. Fertilize fields with hay. Michigan State University Extension, available at https://www.canr.msu.edu/news/fertilize_fields_with_hay.

*Delahunty K. 2015. Investigating Best Practices for the Timing and Amount of Organic Soluble Nitrate Fertigation of Hops in the Northeast. Final Report for FNE14-796. Sustainable Agriculture Research and Education Projects, available at https://projects.sare.org/project-reports/fne14-796/.

*Dixon E, Strik B, Fernandez-Salvador J, DeVetter LW. 2019. Strawberry Nutrient Management Guide for Oregon and Washington. Oregon State University Extension Catalog, available at https://catalog.extension.oregonstate.edu/em9234/html.

*Jarvis-Shean K. 2015. Nitrogen Management for Mature, Bearing Orchards – Research Update. *California Walnuts*, available at https://walnuts.org/resource/nitrogen-management-mature-bearing-orchards-research-update/.

*Van Eerd LL, Congreves KA, Zandstra JW. 2012. Sugar beet (Beta vulgaris L.) storage quality in large outdoor piles is impacted by pile management but not by nitrogen fertilizer or cultivar. Can. J. Plant Sci. 92: 111.

*Sullivan DM, Andrews N, and Brewer LJ. 2020. Estimating Plant-Available Nitrogen Release From Cover Crops. A Pacific Northwest Extension Publication, available at https://catalog.extension.oregonstate.edu/sites/catalog/files/project/pdf/pnw636.pdf.

*Reinprecht Y, Schram L, Marsolais F, Smith TH, Hill B, Pauls KP (2020) Effects of Nitrogen Application on Nitrogen Fixation in Common Bean Production. *Front. Plant Sci.* 11:1172.

**Supplementary Table S4.** Radiocarbon ages and radiocesium peaks and basal (onset) ages included in the ^210^Pb *Plum* model to determine the age-depth relationship of the sediment cores (see Fig. 4 for activity profiles). The error for ^137^Cs is based on the resolution of sampling over depth, as the range between the depth identified as the peak and the next deeper depth sampled. AMS radiocarbon dating was run at the University of Georgia (UGAMS) and Lawrence Livermore National Laboratory (CAMS), and ages are shown as lab reported conventional radiocarbon ages (Stuiver & Polach, 1977) in years before present (present considered 1950 C.E.). ^14^C dates were subsequently calibrated for reservoir correction in the *Plum* model using the IntCal20 Northern Hemisphere calibration curve (Reimer et al., 2020).

| **Site** | **Lab Number** | **Depth (cm)** | **Age ^137^Cs yr C.E.** | **Age ^14^C yr B.P.** |
| --- | --- | --- | --- | --- |
| Harbor | ^137^Cs peak | 21.0 | 1963 ± 20 |  |
| Harbor | ^137^Cs basal | 30.0 | 1953 ± 10 |  |
| Harbor | ^14^C-UGAMS-11115 | 95.0 |  | 690 ± 25 |
| Harbor | ^14^C-UGAMS-11116 | 195.0 |  | 800 ± 25 |
| Harbor | ^14^C-UGAMS-11117 | 230.0 |  | 1420 ± 25 |
| Rubis | ^137^Cs peak | 10.5 | 1963 ± 24 |  |
| Rubis | ^137^Cs basal | 20.5 | 1953 ± 5 |  |
| Rubis | ^14^C-UGAMS-11121 | 45.0 |  | 200 ± 25 |
| Rubis | ^14^C-UGAMS-11122 | 130.0 |  | 480 ± 25 |
| Rubis | ^14^C-UGAMS-11123 | 232.0 |  | 920 ± 25 |
| Yampah | ^137^Cs peak | 24.0 | 1963 ± 3 |  |
| Yampah | ^137^Cs basal | 28.0 | 1953 ± 3 |  |
| Yampah | ^14^C-CAMS-132991 | 75.0 |  | 170 ± 35 |
| Yampah | ^14^C-CAMS-132992 | 180.0 |  | 615 ± 50 |
| Yampah | ^14^C-CAMS-125886 | 275.0 |  | 3090 ± 45 |
| Round Hill | ^137^Cs peak | 15.5 | 1963 ± 16 |  |
| Round Hill | ^137^Cs basal | 29.5 | 1953 ± 10 |  |
| Round Hill | ^14^C-UGAMS-11112 | 100.0 |  | 400 ± 20 |
| Round Hill | ^14^C-UGAMS-11113 | 169.0 |  | 1730 ± 25 |
| Round Hill | ^14^C-UGAMS-11114 | 275.0 |  | 1960 ± 30 |
| Big Creek | ^137^Cs peak | 15.5 | 1963 ± 30 |  |
| Big Creek | ^137^Cs basal | 30.5 | 1953 ± 5 |  |
| Big Creek | ^14^C-UGAMS-11118 | 100.0 |  | 670 ± 25 |
| Big Creek | ^14^C-UGAMS-11119 | 125.0 |  | 2020 ± 25 |
| Big Creek | ^14^C-UGAMS-11120 | 213.0 |  | 2070 ± 20 |
| Azevedo | ^137^Cs peak | 16.0 | 1963 ± 3 |  |
| Azevedo | ^137^Cs basal | 17.0 | 1953 ± 3 |  |
| Azevedo | ^14^C-CAMS-132995 | 70.0 |  | 455 ± 50 |
| Azevedo | ^14^C-CAMS-132994 | 135.0 |  | 1390 ± 40 |
| Azevedo | ^14^C-CAMS-125887 | 192.0 |  | 2315 ± 40 |
| Hudsons | ^137^Cs peak | 15.5 | 1963 ± 16 |  |
| Hudsons | ^137^Cs basal | 21.0 | 1953 ± 5 |  |
| Hudsons | ^14^C-UGAMS-11109 | 75.0 |  | 210 ± 20 |
| Hudsons | ^14^C-UGAMS-11110 | 145.0 |  | 770 ± 20 |
| Hudsons | ^14^C-UGAMS-11111 | 265.0 |  | 1480 ± 25 |

**Supplementary Table S5.** Results of leave-one-out validation to select the variogram model used for Kriging interpolation of point data to create the isoscape and stoichioscape maps (Fig 5). The root mean square error (RMSE) was calculated as the percentage difference between the measured value of a point and the value predicted by an interpolation model that excludes that point. These RMSE values are averages of leave-one-out validation calculated at 15% of the total number of points. Low RSME indicates better prediction by the variogram model. Because of its consistently low RMSE across multiple years, the spherical model was selected for the interpolation maps.

**Average RMSE**

| **Model Type** | **1981 to 2010** | **1839 to 1885** | **1726 to 1839** |
| --- | --- | --- | --- |
| Spherical | 10.5 | 31.0 | 19.9 |
| Circular | 10.6 | 31.0 | 19.9 |
| Exponential | 10.5 | 31.0 | 20.4 |
| Gaussian | 11.1 | 30.4 | 20.5 |
| Linear | 10.6 | 30.9 | 21.3 |
| Linear (linear drift) | 11.5 | 35.4 | 21.2 |
| Linear (quadratic drift) | 18.7 | 48.6 | 26.7 |

**Supplementary Table S6.** Depth-age relationship of the composite chronology including 7 sediment cores and calculated using the ^210^Pb *Plum* model (Fig. 3). Mean years are used as the dates in isoscape and stoichioscape maps.

**Age (Year C.E)**

| **Depth (cm)** | **Midpoint** | **Mean Range** | **Maximum Range** |
| --- | --- | --- | --- |
| 0-10 | 1995 | 1981-2010 | 1977-2010 |
| 10-20 | 1974 | 1963-1981 | 1958-1985 |
| 20-30 | 1959 | 1951-1963 | 1944-1968 |
| 30-40 | 1918 | 1885-1951 | 1851-1958 |
| 40-50 | 1864 | 1839-1885 | 1786-1906 |
| 50-75 | 1782 | 1726-1839 | 1655-1868 |

**Supplementary Table S7.** Raw sediment isotope and stoichiometric data from the 85 grided sediment cores, used as input in the isoscape interpolations.

| East | North | **50 to 75 cm** | | | **40 to 50 cm** | | | **30 to 40 cm** | | | **20 to 30 cm** | | | **10 to 20 cm** | | | **0 to 10 cm** | | |
| --- | --- | --- | --- | --- | --- | --- | --- | --- | --- | --- | --- | --- | --- | --- | --- | --- | --- | --- | --- |
|  |  | δ^15^N | δ^13^C | C/N | δ^15^N | δ^13^C | C/N | δ^15^N | δ^13^C | C/N | δ^15^N | δ^13^C | C/N | δ^15^N | δ^13^C | C/N | δ^15^N | δ^13^C | C/N |
| 608080 | 4072705 | 13.7 | -26.5 | 11.7 | 12.9 | -26.3 | 10.0 | 9.6 | -25.4 | 12.1 | 7.1 | -25.0 | 13.5 | 6.3 | -25.0 | 14.0 | 5.9 | -24.5 | 10.7 |
| 610300 | 4075150 | 7.3 | -27.2 | 13.5 | 7.4 | -25.4 | 11.8 | 7.1 | -24.8 | 8.7 | 6.4 | -24.7 | 8.6 | 4.7 | -23.4 | 13.3 | 3.4 | -22.8 | 14.7 |
| 610300 | 4075350 | 7.4 | -25.0 | 12.5 | 6.1 | -22.0 | 8.3 | 5.9 | -24.6 | 8.8 | 4.8 | -25.1 | 10.2 | 4.2 | -25.1 | 13.9 | 4.5 | -25.5 | 14.0 |
| 610300 | 4075550 | 8.6 | -26.0 | 13.8 | 6.9 | -26.0 | 10.6 | 5.7 | -25.4 | 12.4 | 6.4 | -24.2 | NA | 4.1 | -25.5 | 20.1 | 4.1 | -24.7 | 14.4 |
| 610500 | 4075150 | 6.8 | -25.5 | 12.2 | 6.2 | -25.5 | 11.5 | 4.4 | -24.8 | 11.8 | 4.4 | -24.4 | 11.0 | 4.4 | -24.1 | 13.1 | 3.8 | -25.2 | 14.6 |
| 610500 | 4075350 | 8.1 | -25.8 | 11.5 | 7.1 | -25.0 | 9.9 | 4.5 | -24.3 | 10.7 | 4.8 | -25.3 | 10.5 | 4.3 | -20.4 | 12.3 | 4.1 | -21.1 | 12.2 |
| 610500 | 4075550 | 8.8 | -27.3 | 13.5 | 5.4 | -26.4 | 7.0 | 4.4 | -26.3 | 6.9 | 3.8 | -26.2 | 7.2 | 2.6 | -27.2 | 15.6 | 1.4 | -28.1 | 20.4 |
| 610500 | 4078350 | 10.0 | -26.3 | 13.0 | 0.0 | -27.6 | 15.8 | 0.0 | -24.1 | 12.9 | 6.6 | -25.4 | 11.3 | 7.8 | -26.5 | 14.4 | 6.0 | -25.5 | 13.9 |
| 610500 | 4078550 | 7.6 | -21.7 | 9.2 | 5.9 | -25.1 | 11.8 | 6.2 | -24.5 | 9.8 | 4.7 | -23.0 | 12.2 | 4.6 | -25.0 | 13.1 | 4.0 | -25.4 | 13.1 |
| 610500 | 4078750 | 6.9 | -25.5 | 13.7 | 5.8 | -24.1 | 13.2 | 4.2 | -22.3 | 14.4 | 3.5 | -24.6 | 10.3 | 5.3 | -25.6 | 13.6 | 3.0 | -25.6 | 13.6 |
| 610500 | 4078950 | 5.3 | -24.7 | 14.1 | 5.6 | -25.5 | 10.2 | 4.8 | -25.4 | 10.1 | 5.7 | -25.1 | 9.5 | 2.8 | -25.0 | 15.5 | 5.3 | -23.9 | 15.8 |
| 610500 | 4079150 | 6.1 | -22.5 | 10.3 | 1.8 | -23.0 | 12.6 | 1.5 | -23.9 | 13.2 | 1.7 | -24.5 | 11.6 | 6.3 | -24.5 | 10.2 | 3.5 | -25.4 | 13.2 |
| 610500 | 4079350 | 7.9 | -25.4 | 13.5 | 5.7 | -25.4 | 14.2 | 5.1 | -25.0 | 13.3 | 4.2 | -25.8 | 12.7 | 3.5 | -24.1 | 13.2 | 4.0 | -22.8 | 13.4 |
| 610500 | 4079750 | 8.4 | -26.9 | 13.1 | 4.4 | 0.0 | 19.0 | 4.8 | 0.0 | 12.3 | 3.5 | -25.0 | 19.9 | 3.5 | -22.5 | 16.9 | 3.7 | -23.4 | 16.8 |
| 610700 | 4075150 | 8.8 | -25.9 | 14.1 | 7.0 | -25.7 | 10.9 | 5.5 | -24.0 | 10.4 | 5.7 | -24.5 | 10.5 | 4.9 | -25.2 | 14.8 | 4.8 | -24.6 | 13.8 |
| 610700 | 4075350 | 8.9 | -26.0 | 12.3 | 7.2 | -25.4 | 9.5 | 7.4 | -26.0 | 10.7 | 6.5 | -24.4 | 9.7 | 5.3 | -22.6 | 14.6 | 4.8 | -23.4 | 17.0 |
| 610700 | 4078150 | 6.6 | -24.9 | 13.7 | 5.6 | -21.8 | 13.0 | 4.6 | -23.3 | 11.6 | 4.4 | -25.1 | 11.9 | 3.5 | -25.5 | 17.7 | 4.2 | -24.9 | 17.1 |
| 610700 | 4078350 | 8.4 | -24.8 | 12.3 | 5.3 | 0.0 | 13.4 | 4.9 | -22.2 | 14.4 | 3.0 | -21.9 | 15.3 | 3.6 | -23.8 | 13.0 | 3.5 | -23.4 | 12.3 |
| 610700 | 4078550 | 6.5 | -23.5 | 10.6 | 1.8 | 0.0 | 11.3 | 0.7 | -22.8 | 11.8 | 0.4 | 0.0 | 10.5 | 5.5 | -24.2 | 10.4 | 5.6 | -23.9 | 10.6 |
| 610700 | 4078750 | 6.1 | -23.2 | 13.5 | 5.2 | -23.2 | 10.8 | 5.4 | -23.5 | 10.3 | 5.0 | -23.9 | 10.6 | 4.9 | -24.9 | 14.2 | 5.6 | -24.4 | 13.0 |
| 610700 | 4078950 | 7.0 | -26.2 | 13.0 | 4.4 | -24.2 | 11.0 | 3.4 | -24.9 | 10.2 | 1.9 | -25.0 | 10.2 | 2.5 | -25.8 | 14.2 | 3.0 | -23.9 | 11.4 |
| 610700 | 4079150 | 4.9 | -25.3 | 12.6 | 3.2 | -23.7 | 10.8 | 2.1 | -24.1 | 10.9 | 2.5 | -25.5 | 11.6 | 3.4 | -25.5 | 12.7 | 3.8 | -25.3 | 12.1 |
| 610700 | 4079350 | 8.2 | -26.2 | 13.8 | 6.1 | -24.3 | 10.6 | 4.8 | -24.2 | 12.4 | 4.5 | -24.2 | 11.3 | 3.0 | -25.7 | 12.8 | 3.2 | -24.7 | 14.1 |
| 610700 | 4079950 | 9.4 | -26.4 | 12.7 | 5.2 | -25.4 | 12.1 | 1.8 | -21.7 | 19.4 | -0.1 | -23.5 | 23.2 | 0.7 | -23.0 | 24.2 | 1.1 | -23.3 | 24.8 |
| 610900 | 4075150 | 7.9 | -26.3 | 11.0 | 6.8 | -25.0 | 9.5 | 5.5 | -24.3 | 10.8 | 4.1 | -24.4 | 10.0 | 4.4 | -25.1 | 12.8 | 4.1 | -23.3 | 12.4 |
| 610900 | 4075350 | 5.9 | -27.0 | 14.3 | 5.8 | -26.2 | 6.7 | 4.5 | -23.7 | 7.7 | 4.6 | -24.6 | 24.7 | 5.3 | -24.7 | 11.0 | 3.9 | -22.4 | 11.3 |
| 610900 | 4077950 | 6.0 | -23.1 | 12.3 | 3.3 | 0.0 | 11.0 | 4.3 | 0.0 | 13.2 | 4.1 | -25.7 | 14.1 | 3.9 | -25.9 | 15.8 | 3.7 | -25.2 | 15.5 |
| 610900 | 4078150 | 6.7 | -22.0 | 9.5 | 6.8 | -25.1 | 11.3 | 4.5 | -24.1 | 12.4 | 4.4 | -24.2 | 10.9 | 4.1 | -24.4 | 12.8 | 4.8 | -25.3 | 12.8 |
| 610900 | 4078350 | 7.5 | -25.5 | 13.7 | 7.7 | -24.5 | 10.5 | 6.4 | -24.0 | 11.4 | 4.9 | -24.1 | 12.1 | 4.4 | -24.5 | 13.7 | 3.7 | -24.5 | 13.7 |
| 610900 | 4078550 | 3.8 | -27.1 | 17.1 | 1.6 | -26.8 | 16.8 | 0.6 | -50.8 | 18.8 | 1.1 | -67.4 | 17.5 | 1.3 | -28.4 | 13.0 | 1.1 | -28.4 | 15.9 |
| 610900 | 4078750 | 7.1 | -22.8 | 11.9 | 4.2 | -20.7 | 13.8 | 3.2 | -22.9 | 13.9 | 2.6 | -22.4 | 11.6 | 2.0 | -22.7 | 12.8 | 2.8 | -24.7 | 12.5 |
| 610900 | 4079950 | 8.4 | -27.4 | 14.3 | 3.4 | -26.3 | 11.9 | 2.3 | -26.1 | 14.8 | 2.2 | -26.4 | 13.6 | 1.1 | -27.0 | 17.9 | 0.3 | -27.6 | 27.1 |
| 611100 | 4075150 | 7.6 | -27.6 | 14.1 | 7.6 | -26.1 | 11.3 | 5.6 | -24.4 | 11.3 | 4.4 | -25.4 | 12.1 | 3.9 | -25.1 | 15.4 | 5.4 | -25.7 | 16.4 |
| 611100 | 4075350 | 4.7 | -24.9 | 16.7 | 4.8 | -24.4 | 12.4 | 6.6 | -25.5 | 11.5 | 5.1 | -23.7 | 10.4 | 4.1 | -24.1 | 14.1 | 4.0 | -24.7 | 14.4 |
| 611100 | 4077750 | 6.7 | -25.1 | 12.2 | 3.0 | -25.3 | 13.0 | 5.2 | -24.9 | 12.9 | 4.8 | -25.5 | 12.1 | 8.9 | -24.7 | 10.4 | 5.3 | -24.3 | 13.3 |
| 611100 | 4077950 | 6.4 | -23.2 | 10.8 | 6.7 | -24.0 | 19.3 | 5.0 | -25.5 | 19.0 | 4.5 | -25.1 | 16.4 | 5.1 | -24.7 | 11.1 | 3.9 | -24.3 | 10.1 |
| 611100 | 4078350 | 1.9 | -27.9 | 16.7 | 1.6 | -59.7 | 18.0 | 1.5 | -50.0 | 19.4 | 1.2 | -52.0 | 21.0 | 1.9 | -27.9 | 15.7 | 2.4 | -27.9 | 14.6 |
| 611300 | 4074750 | 7.8 | -25.8 | 12.9 | 7.0 | -24.6 | 11.5 | 5.0 | -24.4 | 13.3 | 2.4 | -24.5 | 12.6 | 4.6 | -24.1 | 12.3 | 4.8 | -25.5 | 13.2 |
| 611300 | 4074950 | 5.8 | -23.2 | 13.2 | 6.8 | -23.6 | 18.6 | 6.2 | -22.9 | 13.1 | 4.6 | -23.3 | 14.9 | 4.8 | -22.1 | 19.2 | 4.6 | -23.3 | 14.4 |
| 611300 | 4075350 | 7.4 | -26.1 | 12.7 | 6.8 | -25.5 | 11.5 | 4.8 | -25.0 | 12.8 | 4.0 | -25.1 | 11.0 | 4.3 | -24.8 | 12.0 | 4.2 | -25.0 | 13.9 |
| 611300 | 4077750 | 6.6 | -21.0 | 9.4 | 5.4 | -25.3 | 11.7 | 3.3 | -25.2 | 11.2 | 3.2 | -25.4 | 11.3 | 2.9 | -25.4 | 14.9 | 3.7 | -23.1 | 11.0 |
| 611300 | 4078150 | 6.0 | -22.7 | 11.9 | 5.7 | -23.7 | 9.3 | 5.3 | -25.5 | 10.2 | 4.3 | -22.3 | 10.9 | 4.3 | -23.1 | 15.2 | 5.5 | -25.6 | 13.9 |
| 611500 | 4074750 | 8.1 | -25.9 | 13.5 | 7.8 | -24.9 | 10.8 | 5.5 | -24.9 | 13.7 | 2.2 | -25.0 | 13.8 | 4.5 | -25.8 | 13.9 | 4.2 | -22.0 | 11.5 |
| 611500 | 4074950 | 7.0 | -22.8 | 10.8 | 0.6 | -24.3 | 10.0 | 1.4 | -23.9 | 10.2 | -0.5 | -23.9 | 11.1 | 5.3 | -24.1 | 12.1 | 4.6 | -23.1 | 13.7 |
| 611500 | 4075350 | 7.7 | -26.3 | 13.8 | 0.0 | 0.0 | 0.0 | 5.7 | -24.7 | 12.7 | 5.2 | -24.3 | 11.9 | 5.8 | -25.6 | 13.6 | 4.8 | -24.9 | 12.7 |
| 611500 | 4077550 | 10.1 | -23.2 | 9.0 | 9.2 | -23.4 | 15.7 | 7.1 | -24.7 | 6.5 | 5.1 | -23.8 | 16.4 | 4.7 | -24.7 | 11.0 | 3.7 | -25.3 | 11.8 |
| 611500 | 4077750 | 9.2 | -25.6 | 11.8 | 6.8 | -25.5 | 12.9 | 4.6 | -23.5 | 13.8 | 2.7 | -21.6 | 13.7 | 3.9 | -24.2 | 12.7 | 3.8 | -23.0 | 12.0 |
| 611500 | 4078150 | 3.0 | -26.7 | 15.0 | 1.9 | -44.8 | 16.6 | 1.5 | -47.8 | 16.3 | 1.4 | -59.3 | 15.8 | 1.3 | -27.9 | 11.2 | 1.1 | -28.1 | 10.9 |
| 611700 | 4074350 | 8.4 | -26.3 | 14.5 | 6.7 | -25.3 | 12.2 | 2.5 | -18.3 | 19.3 | 3.9 | NA | 15.6 | 3.7 | -20.2 | 18.5 | 4.6 | -21.4 | 16.4 |
| 611700 | 4074550 | 8.3 | -26.7 | 13.9 | 7.8 | -26.6 | 13.8 | 7.0 | -25.8 | 13.0 | 5.6 | -25.3 | 13.4 | 4.9 | -25.7 | 12.9 | 5.5 | -25.0 | 12.3 |
| 611700 | 4074750 | 7.9 | -26.9 | 14.4 | 2.7 | -26.0 | 12.2 | 7.2 | -24.2 | 11.2 | 4.3 | -24.3 | 11.1 | 4.7 | -25.1 | 12.5 | 5.9 | -25.7 | 13.7 |
| 611700 | 4074950 | 7.4 | -25.9 | 13.3 | 6.2 | -24.6 | 12.7 | 2.3 | -24.2 | 14.6 | 1.6 | -24.4 | 12.0 | 4.5 | -25.5 | 12.9 | 4.2 | -24.1 | 13.6 |
| 611700 | 4075550 | 8.6 | -25.8 | 13.6 | 3.7 | -26.0 | 15.7 | 3.6 | -23.7 | 13.2 | -0.1 | -24.1 | 10.6 | 5.0 | -25.0 | 12.8 | 5.3 | -24.9 | 13.0 |
| 611700 | 4075950 | 6.1 | -25.4 | 13.4 | 4.5 | -24.9 | 11.8 | 4.9 | -25.7 | 10.9 | 4.3 | -24.3 | 10.0 | 4.2 | -22.9 | 10.7 | 3.4 | -23.2 | 11.0 |
| 611700 | 4076150 | 9.6 | -26.3 | 12.8 | 6.3 | -25.9 | 12.4 | 5.6 | -24.6 | 11.9 | 0.0 | -23.4 | 0.0 | 5.2 | -24.1 | 11.6 | 4.0 | -25.6 | 12.9 |
| 611700 | 4076350 | 7.7 | -26.5 | 13.6 | 6.9 | -27.1 | 13.1 | 6.3 | -26.4 | 12.5 | 4.4 | -25.4 | 11.5 | 4.2 | -25.4 | 11.8 | 4.3 | -25.4 | 14.1 |
| 611700 | 4076550 | 7.5 | -25.6 | 12.4 | 6.6 | -25.7 | 11.3 | 6.4 | -25.7 | 13.8 | 4.7 | -24.9 | 13.5 | 5.1 | -25.1 | 12.5 | 4.8 | -25.3 | 12.9 |
| 611700 | 4077350 | 9.7 | -23.5 | 9.6 | 7.3 | -25.2 | 11.4 | 6.2 | -25.6 | 13.6 | 5.4 | -26.0 | 14.0 | 6.4 | -25.1 | 11.7 | 4.6 | -24.5 | 12.4 |
| 611700 | 4077550 | 10.5 | -25.7 | 11.8 | 9.0 | -26.2 | 10.9 | 8.0 | -26.1 | 10.7 | 6.7 | -26.0 | 11.4 | 5.9 | -25.6 | 13.6 | 5.4 | -25.3 | 12.3 |
| 611700 | 4077750 | 0.2 | -24.9 | 13.9 | 4.2 | -22.2 | 11.7 | 2.1 | -22.1 | 12.8 | 2.7 | -24.0 | 12.4 | 0.4 | -21.4 | 7.9 | 3.9 | -21.9 | 13.8 |
| 611710 | 4075747 | 7.1 | -25.8 | 16.7 | NA | NA | NA | NA | NA | NA | NA | NA | NA | 4.4 | -23.2 | 11.4 | 4.3 | -25.0 | 14.1 |
| 611900 | 4074750 | 9.7 | -25.4 | 12.9 | 7.2 | -25.6 | 12.1 | 6.5 | -25.1 | 12.4 | 4.4 | -25.1 | 17.9 | 5.7 | -24.4 | 12.2 | 4.4 | -24.8 | 12.8 |
| 611900 | 4075550 | 7.1 | -24.7 | 11.7 | 7.8 | NA | NA | 5.9 | NA | NA | 4.2 | NA | NA | 4.0 | -24.2 | 10.9 | 3.7 | -25.2 | NA |
| 611900 | 4076550 | 8.5 | -25.6 | 14.4 | 7.1 | -26.4 | 12.9 | 4.6 | -24.9 | 11.8 | 2.8 | -26.0 | 13.7 | 3.6 | -25.6 | 13.2 | 3.4 | NA | 13.5 |
| 611900 | 4076750 | 7.7 | -25.3 | 12.3 | 7.7 | -24.8 | 11.0 | 7.2 | -24.8 | 12.7 | 6.1 | -25.3 | 12.9 | 3.9 | -25.1 | 12.6 | 4.1 | -23.7 | 12.3 |
| 611900 | 4076950 | 10.1 | -24.7 | 11.9 | 7.4 | -24.7 | 8.9 | 5.9 | -25.1 | 10.4 | 5.2 | -25.5 | 13.9 | 5.4 | -23.7 | 14.3 | 0.0 | -24.7 | 13.7 |
| 611900 | 4077150 | 9.3 | -25.8 | 11.9 | 7.9 | -25.4 | 14.1 | 6.1 | -24.1 | 7.6 | 4.8 | -22.6 | 20.0 | 4.2 | -24.3 | 12.6 | 2.7 | -25.3 | 14.9 |
| 611900 | 4077350 | 6.5 | -23.7 | 12.1 | 6.1 | -25.3 | 7.9 | 4.8 | -25.4 | 8.2 | 4.4 | -25.5 | 18.6 | 1.9 | -25.2 | 13.0 | 4.4 | -23.6 | 11.8 |
| 611900 | 4077750 | 6.3 | -25.2 | 14.2 | 8.2 | -26.3 | 11.6 | 8.2 | -26.3 | 10.9 | 8.2 | -24.4 | 9.8 | 9.3 | -25.7 | 13.1 | 5.5 | -25.0 | 15.0 |
| 611900 | 4077950 | 3.2 | -22.2 | 17.1 | 2.7 | -21.7 | 13.7 | 3.1 | -22.2 | 11.3 | 2.7 | -22.9 | 12.6 | 3.6 | -22.8 | 15.6 | 3.5 | -23.5 | 15.7 |
| 612030 | 4075975 | 8.1 | -24.0 | 10.8 | 7.1 | -25.2 | 12.5 | 5.5 | -24.6 | 13.3 | 5.1 | -25.4 | 13.9 | 6.2 | -25.6 | 15.0 | 4.8 | -23.3 | 12.9 |
| 612040 | 4076175 | 9.5 | -26.0 | 14.9 | 9.1 | -25.2 | 11.5 | 5.7 | -23.7 | 11.8 | 4.6 | -24.8 | 12.5 | 5.6 | -25.7 | 13.5 | 5.0 | -23.8 | 11.8 |
| 612071 | 4076388 | 6.9 | -21.7 | 10.4 | 1.8 | -22.5 | 12.0 | 1.4 | -23.6 | 11.2 | 1.9 | -22.9 | 11.2 | 6.0 | -24.0 | 11.8 | 5.4 | -24.1 | 13.7 |
| 612076 | 4076575 | 7.0 | -22.3 | 11.0 | 0.3 | -23.4 | 11.3 | 0.8 | -23.4 | 11.8 | 1.1 | -23.6 | 11.8 | 5.6 | -23.6 | 12.3 | 6.1 | -23.5 | 12.0 |
| 612100 | 4075550 | 7.6 | -24.8 | 12.4 | 7.5 | -24.0 | 10.3 | 5.6 | -23.4 | 10.3 | 4.1 | -24.3 | 16.7 | 4.9 | -24.7 | 12.6 | 4.2 | -25.2 | 15.7 |
| 612100 | 4075750 | 8.8 | -25.1 | 12.3 | 7.0 | -24.2 | 12.2 | 5.9 | -24.2 | 12.5 | 4.3 | -24.3 | 12.8 | 5.1 | -24.8 | 12.1 | 4.1 | -25.6 | 13.6 |
| 612100 | 4076950 | 8.4 | -25.6 | 12.8 | 5.0 | -21.9 | 9.9 | 5.0 | -25.2 | 13.6 | 2.6 | -24.6 | 13.8 | 4.2 | -25.6 | 12.3 | 4.0 | -25.4 | 13.3 |
| 612100 | 4077150 | 9.4 | -24.8 | 12.9 | 7.5 | -25.6 | 11.7 | 6.5 | -25.2 | 12.3 | 5.1 | -24.3 | 12.2 | 4.5 | -25.1 | 14.0 | 4.4 | -24.6 | 13.5 |
| 612100 | 4077350 | 6.7 | -22.5 | 9.8 | 2.5 | -23.2 | 12.0 | 2.2 | 0.0 | 10.8 | 1.2 | -24.0 | 12.5 | 5.9 | -23.2 | 10.6 | 4.9 | -24.4 | 12.2 |
| 612100 | 4077550 | 8.3 | -21.8 | 9.3 | 3.0 | -22.5 | 11.1 | 3.3 | -26.9 | 11.1 | 2.8 | -23.2 | 12.2 | 6.2 | -22.6 | 9.7 | 5.5 | -23.7 | 10.3 |
| 612300 | 4076750 | 0.9 | -28.1 | 29.9 | 5.5 | -38.5 | 21.8 | 1.1 | -24.0 | 19.1 | 1.6 | -26.9 | 19.2 | 2.3 | -27.2 | 22.2 | 1.8 | -27.4 | 22.3 |
| 612300 | 4076950 | 5.7 | -22.4 | 13.0 | 1.9 | -22.9 | 12.9 | 5.0 | -24.9 | 12.3 | 3.6 | -23.6 | 13.5 | 3.4 | -22.1 | 15.6 | 2.4 | -25.9 | 18.6 |
| 612300 | 4077150 | 5.1 | -24.3 | 13.7 | 2.2 | -24.1 | 13.2 | 1.7 | -24.8 | 13.1 | 2.3 | -24.5 | 14.3 | 4.5 | -24.8 | 14.9 | 5.0 | -24.5 | 14.0 |
| 612300 | 4077350 | 7.4 | -25.3 | 14.7 | 4.4 | -24.5 | 12.1 | 3.7 | -24.5 | 12.1 | 3.4 | -24.0 | 11.3 | 4.1 | -24.7 | 12.9 | 4.9 | -24.5 | 13.0 |
| 612300 | 4077550 | 6.8 | -21.9 | NA | 2.9 | -24.9 | NA | 5.0 | -24.6 | NA | 3.2 | -25.4 | NA | 2.8 | -25.9 | 13.6 | 2.6 | -25.6 | NA |

**Supplementary Table S8.** Raw sediment isotope and stoichiometric data from the 6 high-resolution sediment cores used for timeseries analysis of trends.

| Depth  (cm) | **Harbor** | | | **Rubis** | | | **Yampah** | | | **Round Hill** | | | **Big Creek** | | | **Hudsons** | | |
| --- | --- | --- | --- | --- | --- | --- | --- | --- | --- | --- | --- | --- | --- | --- | --- | --- | --- | --- |
|  | δ^15^N | δ^13^C | C/N | δ^15^N | δ^13^C | C/N | δ^15^N | δ^13^C | C/N | δ^15^N | δ^13^C | C/N | δ^15^N | δ^13^C | C/N | δ^15^N | δ^13^C | C/N |
| 1 | 14.8 | -26.6 | 12.0 | 7.0 | -24.1 | 14.3 | 8.0 | -25.9 | 11.8 | 7.9 | -25.7 | 13.9 | 10.5 | -24.9 | 11.4 | 9.8 | -26.1 | 12.6 |
| 2.5 | 14.9 | -26.7 | 11.6 | 6.8 | -24.3 | 15.1 | NA | NA | NA | 7.1 | -25.8 | 16.0 | 9.8 | -25.4 | 11.6 | 9.0 | -26.3 | 13.6 |
| 3.5 | 14.5 | -26.3 | 12.0 | 6.4 | -24.7 | 16.1 | NA | NA | NA | 7.4 | -26.1 | 15.4 | 9.8 | -25.5 | 11.5 | 8.9 | -26.5 | 13.6 |
| 4.5 | 14.1 | -26.6 | 11.7 | 6.7 | -24.5 | 13.7 | NA | NA | NA | 7.9 | -25.9 | 14.7 | 9.6 | -26.4 | 12.8 | 9.0 | -27.0 | 14.3 |
| 5.5 | 13.6 | -26.9 | 12.0 | 6.5 | -24.4 | 15.5 | 6.8 | -25.8 | 10.2 | 6.4 | -25.6 | 16.1 | 9.5 | -26.4 | 12.2 | 8.5 | -27.0 | 14.0 |
| 6.5 | 13.7 | -26.0 | 10.9 | 6.8 | -24.9 | 14.6 | NA | NA | NA | 6.4 | -25.1 | 15.2 | 9.2 | -26.3 | 12.5 | 8.3 | -26.3 | 14.4 |
| 7.5 | 12.5 | -26.8 | 10.9 | 6.5 | -24.9 | 14.2 | NA | NA | NA | 6.6 | -25.9 | 14.6 | 8.8 | -26.4 | 12.6 | 7.9 | -26.2 | 13.0 |
| 8.5 | 12.4 | -26.4 | 12.2 | NA | NA | NA | NA | NA | NA | 6.6 | -25.5 | 15.1 | 8.6 | -26.3 | 12.3 | 7.6 | -26.2 | 12.5 |
| 9.5 | 12.9 | -26.2 | 11.2 | 6.9 | -24.8 | 14.9 | NA | NA | NA | 6.8 | -25.8 | 14.9 | 8.1 | -26.1 | 12.1 | 7.5 | -26.2 | 12.9 |
| 10.5 | 13.1 | -26.6 | 11.9 | 7.2 | -25.2 | 13.5 | 7.4 | -26.5 | 15.0 | 7.0 | -25.7 | 14.6 | 8.1 | -26.4 | 12.4 | 6.8 | -25.9 | 14.3 |
| 11.5 | NA | NA | NA | 7.1 | -25.0 | 15.7 | NA | NA | NA | 6.8 | -25.5 | 14.3 | 8.0 | -25.4 | 12.3 | 6.3 | -25.6 | 13.4 |
| 12.5 | 12.7 | -26.7 | 10.9 | NA | NA | NA | NA | NA | NA | 6.8 | -26.5 | 14.6 | 8.5 | -26.0 | 12.3 | 6.0 | -25.5 | 13.5 |
| 13.5 | 13.1 | -26.5 | 11.0 | 6.7 | -25.3 | 14.7 | NA | NA | NA | NA | NA | NA | NA | NA | NA | NA | NA | NA |
| 14.5 | 12.9 | -26.4 | 11.6 | 6.8 | -25.3 | 14.4 | NA | NA | NA | 6.0 | -25.9 | 14.3 | 8.1 | -26.1 | 12.7 | 4.8 | -25.1 | 13.5 |
| 15.5 | 12.6 | -26.2 | 11.8 | 6.4 | -25.4 | 15.3 | 7.8 | -25.9 | 13.4 | 5.8 | -25.4 | 14.2 | 8.1 | -25.8 | 12.9 | 4.3 | -24.7 | 13.9 |
| 16.5 | 13.1 | -26.2 | 11.1 | 6.3 | -25.0 | 14.3 | NA | NA | NA | 5.3 | -25.0 | 14.6 | 7.6 | -25.1 | 12.6 | NA | NA | NA |
| 17.5 | 13.0 | -26.2 | 12.0 | 6.3 | -25.0 | 14.0 | NA | NA | NA | 5.3 | -25.2 | 14.9 | 7.8 | -25.1 | 13.1 | 3.3 | -22.0 | 16.9 |
| 18.5 | 12.7 | -25.8 | 11.6 | 6.1 | -25.3 | 14.6 | NA | NA | NA | 5.1 | -25.7 | 15.0 | NA | NA | NA | 2.9 | -22.2 | 16.6 |
| 19.5 | 12.9 | -25.7 | 11.9 | NA | NA | NA | NA | NA | NA | 4.9 | -24.6 | 14.8 | 7.2 | -25.4 | 13.0 | 2.5 | NA | 18.5 |
| 20.5 | NA | NA | NA | NA | NA | NA | 5.9 | -24.9 | 12.5 | NA | NA | NA | 7.0 | -24.7 | 12.6 | 2.2 | NA | 19.3 |
| 21.5 | 11.3 | -25.6 | 11.3 | NA | NA | NA | NA | NA | NA | 3.6 | -23.6 | 14.7 | 6.8 | -25.1 | 13.0 | 2.1 | -21.6 | 20.2 |
| 22.5 | 10.9 | -25.5 | 11.8 | NA | NA | NA | NA | NA | NA | 3.7 | -23.6 | 14.3 | NA | NA | NA | 2.0 | NA | 20.5 |
| 23.5 | 9.9 | -25.4 | 14.5 | 5.9 | -25.3 | 15.2 | NA | NA | NA | 3.8 | -23.2 | 14.0 | 6.4 | -25.2 | 13.9 | 2.0 | -20.4 | 20.7 |
| 24.5 | 10.0 | -25.4 | 14.7 | 5.4 | -25.0 | 13.8 | NA | NA | NA | 3.7 | -23.7 | 16.1 | 6.1 | -25.5 | 14.2 | 2.0 | -20.4 | 20.5 |
| 25.5 | 9.1 | -25.4 | 14.7 | 5.6 | -25.4 | 13.6 | 4.7 | -27.5 | 18.2 | 3.3 | -21.2 | 16.8 | 5.7 | -24.8 | 14.4 | 1.2 | -20.6 | 21.0 |
| 26.5 | 8.9 | -25.5 | 14.8 | 5.3 | -25.1 | 13.6 | NA | NA | NA | 3.4 | -22.5 | 16.5 | 6.0 | -25.0 | 14.5 | 1.9 | NA | 20.7 |
| 27.5 | NA | NA | NA | 5.2 | -24.9 | 13.6 | NA | NA | NA | 3.2 | -21.7 | 18.3 | 5.7 | -25.0 | 13.5 | 1.7 | -20.1 | 21.3 |
| 28.5 | 8.6 | -25.4 | 15.4 | 5.0 | -24.3 | 14.2 | NA | NA | NA | 3.4 | NA | 17.8 | 5.5 | -25.0 | 13.3 | 1.8 | -20.7 | 21.2 |
| 29.5 | 8.2 | -25.5 | 16.4 | 4.8 | -24.7 | 14.2 | NA | NA | NA | 3.2 | NA | 18.6 | 5.6 | -24.5 | 12.7 | 1.6 | -21.3 | 21.6 |
| 30.5 | 8.8 | -25.6 | 15.4 | 4.9 | -24.6 | 13.8 | 3.4 | -23.8 | 11.9 | 2.9 | NA | 18.3 | NA | -24.7 | 12.6 | 1.2 | -20.4 | 21.2 |
| 31.5 | 7.2 | -24.5 | 15.8 | 4.6 | -24.3 | 13.5 | NA | NA | NA | 2.9 | -21.1 | 18.2 | 5.2 | -24.0 | 12.9 | 0.8 | -20.3 | 21.7 |
| 32.5 | 6.7 | -24.5 | 17.0 | 4.7 | -24.3 | 13.7 | NA | NA | NA | 2.5 | NA | 18.4 | 4.8 | -23.2 | 13.1 | 1.0 | NA | 20.8 |
| 33.5 | 6.7 | -25.5 | 14.6 | 4.6 | -24.3 | 12.8 | NA | NA | NA | 2.6 | -20.5 | 18.2 | 4.4 | -23.2 | 12.7 | 1.3 | -20.2 | 21.8 |
| 34.5 | 6.5 | -24.9 | 15.6 | 4.7 | -23.6 | 13.8 | NA | NA | NA | 2.6 | -21.3 | 17.6 | 5.0 | -22.6 | 12.0 | 1.3 | -20.6 | 22.3 |
| 35.5 | 7.0 | -24.1 | 16.4 | 4.6 | -23.9 | 12.9 | 4.3 | -22.4 | 12.2 | 3.0 | -25.9 | 18.1 | 4.8 | -21.8 | 11.7 | 0.6 | -20.5 | 22.9 |
| 36.5 | 7.4 | -24.8 | 15.8 | 4.9 | -24.2 | 12.4 | NA | NA | NA | 2.9 | -25.8 | 17.4 | 4.9 | -21.3 | 11.9 | 0.5 | -20.4 | 24.1 |
| 37.5 | 6.6 | -24.9 | 15.6 | 4.7 | -24.2 | 12.2 | NA | NA | NA | 3.0 | -26.5 | 16.4 | 4.7 | -20.8 | 11.6 | 0.9 | -20.9 | 23.4 |
| 38.5 | 7.2 | -24.6 | 15.7 | 4.9 | NA | 12.9 | NA | NA | NA | 2.9 | -26.6 | 17.8 | 4.9 | -21.2 | 12.2 | 0.4 | -20.7 | 25.0 |
| 39.5 | 6.8 | -23.7 | 15.2 | 5.1 | -24.3 | 11.6 | NA | NA | NA | 3.1 | -26.9 | 15.8 | 4.6 | -21.1 | 12.5 | 0.2 | -20.8 | 25.2 |
| 40.5 | 7.1 | -24.6 | 13.7 | 5.4 | NA | 11.2 | 4.1 | -23.0 | 11.9 | 3.0 | -26.9 | 16.4 | 4.8 | -21.0 | 12.0 | 0.0 | -21.2 | 24.8 |
| 41.5 | 6.6 | -24.5 | 15.4 | 5.6 | -24.0 | 11.9 | NA | NA | NA | 3.0 | -27.1 | 16.1 | 4.8 | -22.2 | 11.5 | 0.1 | -21.0 | 25.5 |
| 42.5 | 6.8 | -24.8 | 15.7 | NA | NA | NA | NA | NA | NA | 2.8 | -27.0 | 16.5 | 4.3 | -21.6 | 12.8 | 0.2 | -21.3 | 25.5 |
| 43.5 | 6.9 | -24.7 | 12.6 | NA | NA | NA | NA | NA | NA | 3.2 | -27.0 | 15.1 | 4.5 | -23.0 | 12.2 | NA | NA | NA |
| 44.5 | 6.8 | -25.2 | 12.7 | NA | NA | NA | NA | NA | NA | 3.2 | -27.1 | 15.6 | 4.5 | -22.2 | 12.3 | NA | NA | NA |
| 45.5 | 7.6 | -25.5 | 15.2 | NA | NA | NA | 2.9 | -21.2 | 16.2 | NA | NA | NA | 4.3 | -23.7 | 14.3 | NA | NA | NA |

**Supplementary Table S9.** Citations for images from IAN (Fig. 1). Available at: ian.umces.edu/media-library.

| **Image Title** | **Author** | **Company** | **Date Created** |
| --- | --- | --- | --- |
| *Quercus bicolor*  (Swamp White Oak) | Annie Carew | Integration and Application Network | 2022-02-09 |
| *Distichlis spicata*  (Coastal Salt Grass) | [Jane Hawkey](https://ian.umces.edu/about/who-we-are/jane-hawkey/) | Integration and Application Network | 2012-06-01 |
| *Tecticornia* spp.  (Samphire) | Dieter Tracey | Marine Botany UQ | 1999-01-01 |
| Phytoplankton bloom | Diana Kleine | Marine Botany UQ | 2001-06-12 |
| *Ulva* spp. (Sea Lettuce) | Tacey Saxby | Integration and Application Network | 2005-08-11 |
| Water: septic truck icon | Jane Hawkey | Integration and Application Network | 2016-05-01 |
| Fertilizer | Jane Hawkey | Integration and Application Network | 2013-09-26 |
